# A Systematic Health Assessment of Indian Ocean Bottlenose (*Tursiops aduncus*) and Indo-Pacific Humpback (*Sousa plumbea*) Dolphins Incidentally Caught in Shark Nets off the KwaZulu-Natal Coast, South Africa

**DOI:** 10.1371/journal.pone.0107038

**Published:** 2014-09-09

**Authors:** Emily P. Lane, Morné de Wet, Peter Thompson, Ursula Siebert, Peter Wohlsein, Stephanie Plön

**Affiliations:** 1 Department of Research and Scientific Services, National Zoological Gardens of South Africa, Pretoria, South Africa; 2 Epidemiology Section, Department of Production Animal Studies, Faculty of Veterinary Science, University of Pretoria, Pretoria, South Africa; 3 Institute for Terrestrial and Aquatic Wildlife Research, University of Veterinary Medicine, Hannover, Foundation, Germany; 4 Department of Pathology, University of Veterinary Medicine, Hannover, Foundation, Germany; 5 South African Institute for Aquatic Biodiversity, c/o Port Elizabeth Museum/Bayworld, Port Elizabeth, South Africa; Veterinary Pathology, Switzerland

## Abstract

Coastal dolphins are regarded as indicators of changes in coastal marine ecosystem health that could impact humans utilizing the marine environment for food or recreation. Necropsy and histology examinations were performed on 35 Indian Ocean bottlenose dolphins (*Tursiops aduncus*) and five Indo-Pacific humpback dolphins (*Sousa plumbea*) incidentally caught in shark nets off the KwaZulu-Natal coast, South Africa, between 2010 and 2012. Parasitic lesions included pneumonia (85%), abdominal and thoracic serositis (75%), gastroenteritis (70%), hepatitis (62%), and endometritis (42%). Parasitic species identified were *Halocercus* sp. (lung), *Crassicauda* sp. (skeletal muscle) and *Xenobalanus globicipitis* (skin). Additional findings included bronchiolar epithelial mineralisation (83%), splenic filamentous tags (45%), non-suppurative meningoencephalitis (39%), and myocardial fibrosis (26%). No immunohistochemically positive reaction was present in lesions suggestive of dolphin morbillivirus, *Toxoplasma gondii* and *Brucella* spp. The first confirmed cases of lobomycosis and sarcocystosis in South African dolphins were documented. Most lesions were mild, and all animals were considered to be in good nutritional condition, based on blubber thickness and muscle mass. Apparent temporal changes in parasitic disease prevalence may indicate a change in the host/parasite interface. This study provided valuable baseline information on conditions affecting coastal dolphin populations in South Africa and, to our knowledge, constitutes the first reported systematic health assessment in incidentally caught dolphins in the Southern Hemisphere. Further research on temporal disease trends as well as disease pathophysiology and anthropogenic factors affecting these populations is needed.

## Introduction

Surveillance and research on diseases in wildlife populations present many challenges but are important tools to identify changes in ecosystem health and emerging threats to human and animal health [1]. Health assessments in coastal cetaceans can be used to indirectly monitor marine ecosystem health, investigate the effects of human activities on animal health, and identify risks to humans utilizing the same habitat for food or recreation [2], [3]. Marine mammal researchers over the past 40 years have raised concerns about deteriorating ocean health. Although increased surveillance and improved diagnostic techniques may account for a portion of the recent proliferation of disease reports [4], mortality events due to harmful algal blooms and morbillivirus outbreaks are thought to be increasingly common in the North Atlantic [4]–[6]. However, lack of baseline data precludes accurate recording of temporal changes in the prevalence of many diseases [4], [7], [8]. Expected increasing effects of climate change, inter- and intra-specific competition and habitat degradation as well as exposure to pollutants, lend new urgency to understanding the causes of marine mammal disease outbreaks [3], [7]–[9].

Coastal cetaceans are particularly vulnerable to anthropogenic impacts including net entanglement [10], boat strike [11], disturbances due to boat traffic [10], pollution [7], nutrient enrichment [10], novel pathogens [12], habitat degradation [10], and prey depletion through fishing [10], [12]. Dolphins have long life spans [12], [13], feed at a high trophic level [13], and their fat stores accumulate chemical pollutants [13]–[15]. Increased mortalities in polluted waters during morbillivirus epidemics suggest that pollutants may impair disease defense mechanisms [12]. Habitat destruction and prey depletion increase inter- and intra-species competition and stress that further undermine host defense mechanisms [7], [12]. Nutrient enrichment with sewage and fertilizers has been implicated in an increase in the occurrence of devastating toxic algal blooms [16], [17]. River runoff from urban areas may be responsible for the introduction of new marine pathogens such as *T. gondii* [18], [19].

Both *Tursiops aduncus* (Indian Ocean bottlenose dolphin) and *Sousa plumbea* (Indo-Pacific humpback dolphin) occur along the Southern African coast within 10 km of the shore, [20]–[23]. Gill nets are deployed off the South African east coast by the KwaZulu-Natal Sharks Board (KZNSB) to reduce the risk of shark-human interactions [22], [24]. Approximately 20 dolphins, mainly *T. aduncus* and *S. plumbea*, are incidentally caught (by-caught) annually in the shark nets [25]. This paper reports the results of the first systematic health assessment of incidentally caught coastal dolphins, based on 40 animals examined between 2010 and 2012. Pathological findings are analyzed in relation to species, catch location, age, sex, and body condition. This survey provides valuable baseline data for assessing the health status of these dolphin populations and for future monitoring of temporal and spatial health trends.

## Materials And Methods

### Ethics Statement

Evaluation of dolphins incidentally caught in the shark nets was performed under research permits issued to the Port Elizabeth Museum/Bayworld (PEM) by the South African Departments of Environmental Affairs and Agriculture, Forestry and Fisheries (RES2012/40 and RES2013/19). The protocol for this study was approved by the Research Committee of the Faculty of Veterinary Science; the Animal Use and Care Committee of the University of Pretoria (Protocol V011/12) and the Ethics and Scientific Committee of the National Zoological Gardens of South Africa (P10/23). Formalin-fixed tissues are stored at the PEM; paraffin embedded tissues and glass slides are stored at the National Zoological Gardens of South Africa.

From April 2010 to April 2012, dead dolphins were retrieved from the shark nets, weighed and frozen at -20°C by the KZNSB. Every 6–8 months, carcasses were defrosted and morphological measurements taken [26]. Of the 46 dolphins retrieved, 35 *T. aduncus* and five *S. plumbea* were deemed sufficiently fresh for necropsy and histopathological examination [27]. Age was estimated by total body length in *T. aduncus* [21] and by counting the annual growth layers in a mandibular tooth in *S. plumbea*. Animals were classified as unweaned calves (<2 years), juveniles (2–12 years), or sexually mature adults (>12 years) [21], [28]. Blubber thickness measurements were used (ventral, lateral and dorsal midline cranial to the dorsal fin) to assess nutritional condition [29].

Using a standard necropsy and sampling protocol [30], all organs were examined macroscopically and representative samples fixed in 10% buffered formalin. Paraffin wax embedded tissues were sectioned (5 µm) and stained with haematoxylin and eosin (HE). Selected tissues were also stained with Gram, Von Kossa (VK), Stamps, Masson's Trichrome (MT), Ziehl-Neelsen (ZN), Gomori's methenamine silver (GMS), Perl's prussian blue, Hall's bile, periodic acid-Schiff (PAS), Fontana Masson's and Bielschowsky's modified silver stains [31]. Immunohistochemical reactions for *Toxoplasma gondii* (Department of Pathology, University of Pretoria) and dolphin morbillivirus (Department of Pathology, University of Veterinary Medicine, Hannover) [33] were performed on sections where lymphoplasmacytic inflammation was present in the brain, lung, muscle or heart.

Parasites found during necropsy were preserved in 70% ethanol and identified according to published methods [34]. Lung tissue samples from all 40 dolphins were frozen, until the end of the collection period, thawed in the laboratory and cultured using standard bacteriological methods.

### Statistical analyses

**Figure 1:**
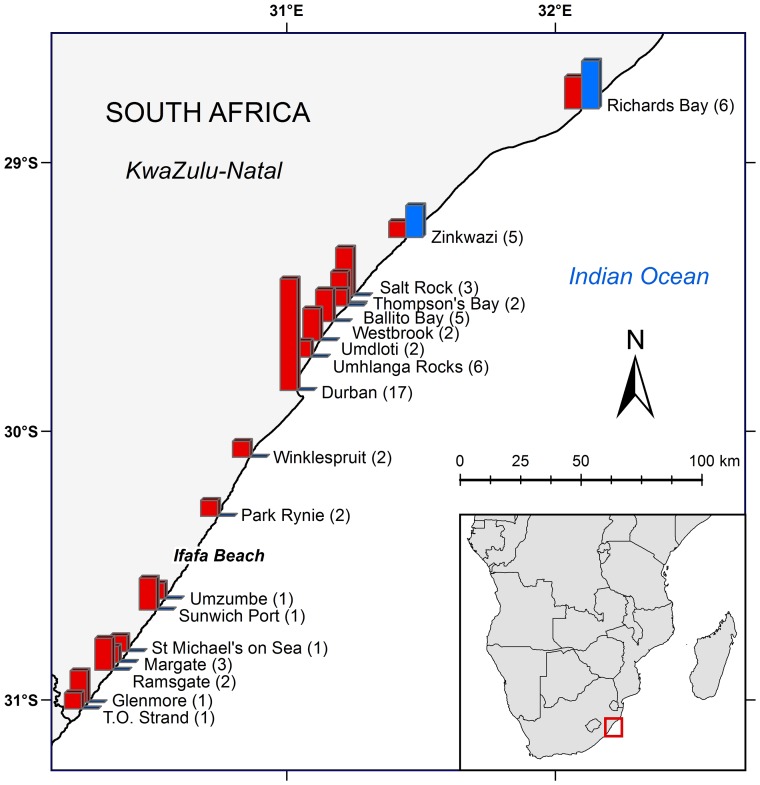
Location (beach name), number of shark nets per beach (in parenthesis) and number of *T. aduncus* (red) and *S. plumbea* (blue) sampled along the KwaZulu-Natal coast, South Africa. Gill nets are 110 m long and 10 m deep. Adapted from [87].

Animals were divided into two groups based on capture location region: North and South of Ifafa beach (Figure 1), since population and genetic studies of *T. aduncus* indicate that these are different subpopulations [35], [36]. Too few *S. plumbea* were sampled for statistical analysis; all statistical comparisons are for *T. aduncus* only, unless otherwise stated. Blubber thickness was compared between age classes and sample sites using a linear mixed model adjusted for sex and region with Bonferroni correction for multiple comparisons. Occurrence of selected lesions with possible biological significance was compared between species, and for *T. aduncus*, between age classes, sexes and capture location region using Fisher's exact test. For univariable associations with p<0.25, adjustment for possible confounding between age class, sex and region was done using multivariable exact logistic regression models. Associations between the occurrence of selected lesions within the same animals was tested using McNemar's test. Due to the exploratory nature of the analysis and the relatively small sample size, significance was assessed at p<0.1. Statistical analysis was done using Stata 12.1 (StataCorp, College Station, TX, U.S.A.).

## Results

More *T. aduncus* (35; 88%) were caught in the nets than *S. plumbea* (5; 12%) (Figure 1). Most *T. aduncus* (25; 71%) and all five *S. plumbea* were sampled from the northern region nets; and seven of the 35 *T. aduncus* (20%) were from the nets off Durban. Most *T. aduncus* in all age classes were females (24; 69%); and more juveniles (16; 46%) and calves (11; 31%) were caught than adults (8; 22%) of both sexes.

**Figure 2:**
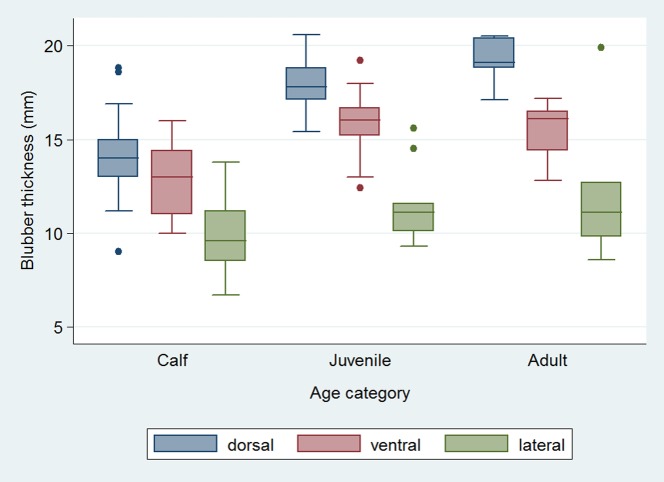
Blubber thickness (mm) of *T. aduncus* in three age classes. Box extends from 25th to 75th percentile, horizontal line represents the median, whiskers extend to the smallest and largest observations that are <1.5 times removed from the interquartile range (IQR), and dots represent outliers.

Blubber was thicker at the dorsal and thinner at the lateral sampling site for each age class (p<0.05; Figure 2). Blubber thickness did not differ between the sexes or between dolphins from different regions. Blubber was thicker in juveniles and adults compared to calves, at the dorsal (p<0.001) and ventral (p<0.05) sites.

Moderate to severe autolysis, putrefaction and freezing artefact were present histologically in most organs, particularly in the respiratory and intestinal mucosae, pancreas, brain and eye. Eosinophils were relatively well preserved compared to other inflammatory cells. Freezing distorted tissue architecture and caused lysis of erythrocytes. In addition, variable numbers of variably sized, round to oval, vacuoles (<0.1 cm diameter) with no associated nuclei or saprophytic bacteria were found in blood vessel lumina and the parenchyma of various organs. Mild to severe, acute congestion was present in most organs in all the dolphins.

Dolphin number, species, sex, age, sampling region, lesion severity and health status for *T. aduncus* and *S. plumbea* are listed in Table S1. Common and newly reported lesions and lesions that may have affected organ function are described below, along with their prevalence in *T. aduncus* and *S. plumbea* (Table 1). Exact logistic regression models for lesions significantly associated (p<0.1) with age class, sex and region are given in Table 2. Supplementary materials include a complete list, with prevalence by species, age class and region, of all pathological findings (Table S2) and common pathology observed in *T. aduncus* by age class, sex and region (Table S3).

**Table 1:** Common pathology observed in Indian Ocean bottlenose (*Tursiops aduncus*) and Indo-Pacific humpback (*Sousa plumbea*) dolphins incidentally caught in shark nets, and bivariable association with species.

Lesion/abnormality	Total (%)	Species (*n*)		
		*T. aduncus*	*S. plumbea*	p*
Combined pneumonia	93	32/35	5/5	1.000
Bronchopneumonia	18	7/35	0/5	0.565
Interstitial pneumonia	63	22/35	3/5	1.000
Broncho-interstitial pneumonia	30	9/35	3/5	0.149
Pulmonary parasites	15	6/35	0/5	1.000
Pleuritis	30	10/35	2/5	0.627
Bronchiolar mucosal calcification	83	29/35	4/5	1.000
Pulmonary anthracosis	8	2/35	1/5	0.338
Gastritis all compartments	68	24/34	2/4	0.577
First and second compartment gastritis	63	23/34	1/4	0.132
Third compartment gastritis	65	14/21	1/2	1.000
Parasitic nodules all compartments	32	12/34	0/4	0.556
Parasitic nodules in the first and second gastric compartments	8	3/34	0/4	1.000
Parasitic nodules in the third gastric compartment	43	10/21	0/2	0.486
Pyloric mucosal calcification	26	5/21	1/2	0.462
Enteritis	68	25/35	2/5	0.307
Periportal hepatitis	54	21/35	0/4	**0.037**
Hepatic serositis	23	9/35	0/4	0.556
Periportal fibrosis	26	9/35	1/4	1.000
Hepatic trematode eggs	8	3/35	0/4	1.000
Bile ductular hyperplasia	44	15/35	2/4	1.000
Splenic filamentous peritonitis	45	17/35	1/5	0.355
Splenic serositis	28	11/35	0/5	0.298
Cervical lymph node serositis	26	10/34	0/5	0.302
Mesenteric lymphnode serositis	46	15/34	3/5	0.647
Marginal lymph node serositis	43	11/27	2/3	0.565
Marginal lymph node anthracosis	10	3/27	0/3	1.000
Endometritis	42	10/24	1/2	1.000
Metritis	23	5/24	1/2	0.415
Oophoritis	19	4/24	1/2	0.354
Mastitis	43	3/7	-	-
Mammary corpora amylacea	43	3/7	-	-
Testicular serositis	38	3/10	2/3	0.510
Endo-, myo- and epicarditis	51	20/35	0/4	**0.047**
Cardiac fibrosis	26	9/35	1/4	1.000
Meningoencephalitis	39	7/16	0/2	0.497
Myositis	19	6/32	1/5	1.000
Combined serositis	75	26/35	4/5	1.000
Abdominal serositis	60	20/35	4/5	0.631
Thoracic serositis	20	18/35	2/5	1.000

*Fisher's exact test; statistically significant results (p<0.100) in bold.

**Table 2:** Associations of age, sex and region with presence of various lesions in *T. aduncus*: results of multivariable exact logistic regression models.

Variable and level		Age class			Sex	Region
		Calf (<2 y)	Juvenile (2–12 y)	Adult (>12 y)	male vs. female	south vs. north
Pleuritis	OR^1^	1*	1.54	0.26	**6.50**	1.17
	95% C.I.^2^	-	0.19, 13.65	0.00, 2.53	**0.98, 59.17**	0.00, 11.33
	p*	-	0.952	0.270	**0.053**	1.000
Pulmonary pneumoconiosis	OR	1*	1.00	**9.52**	1.50	3.00
	95% C.I.	-	0.00, ∞	**0.72, ∞**	0.04, ∞	0.08, ∞
	p	-	–	**0.085**	0.800	0.500
Enteritis	OR	1*	**15.26** *	**6.55**	0.33	0.17
	95% C.I.	-	**1.95, ∞**	**0.82, ∞**	0.02, 3.78	0.00, 2.48
	p	-	**0.006**	**0.080**	0.573	0.303
Gastritis	OR	1*	5.66	**6.21**	1.13	1.97
	95% C.I.	–	0.57, 291.4	**0.78, ∞**	0.14, 9.89	0.23, 26.05
	p	–	0.201	**0.090**	0.141	0.785
Gastritis	OR	1*	7.38	**7.02**	1.36	1.09
(compartments 1&2)	95% C.I.	–	0.76, 376.8	**0.89, ∞**	0.17, 10.91	0.12, 10.17
	p	–	0.104	**0.066**	1.000	1.000
Periportal fibrosis	OR	1*	3.02	**12.64**	1.21	1.71
	95% C.I.	–	0.30, 41.55	**1.17, 223.9**	0.13, 9.89	0.18, 15.94
	p	–	0.482	**0.033**	1.000	0.884
Splenic tags	OR	1*	2.20	4.33	2.01	**7.75**
	95% C.I.	–	0.29, 18.27	0.42, 67.24	0.33, 14.21	**1.10, 99.82**
	p	–	0.607	0.300	0.621	**0.037**
Splenic serositis	OR	1*	2.81	5.41	**11.07**	3.3
	95% C.I.	–	0.27, 40.96	0.37, 117.1	**1.51, 152.0**	0.36, 45.61
	p	–	0.553	0.307	**0.012**	0.408
Cervical lymph node serositis	OR	1*	1.42	4.77	**7.42**	3.90
	95% C.I.	–	0.13, 15.20	0.36, 90.18	**1.04, 95.28**	0.45, 54.29
	p	–	1.000	0.327	**0.045**	0.297
Mesenteric lymph node serositis	OR	1*	2.85	**16.82**	3.56	0.94
	95% C.I.	–	0.42, 23.36	**1.92, ∞**	0.53, 29.82	0.10, 7.54
	p	–	0.377	**0.009**	0.247	1.000
Endometritis	OR	1*	0.92	**8.10**	–	0.92
	95% C.I.	–	0.07, 9.14	**0.87, ∞**	–	0.07, 9.14
	p	–	1.000	**0.067**	–	1.000
Cardiac fibrosis	OR	1*	**13.97**	**51.63**	4.29	0.71
	95% C.I.	–	**1.54, ∞**	**5.35, ∞**	0.26, 280.2	0.04, 13.09
	p	–	**0.017**	**0.001**	0.498	1.000
Myositis	OR	1*	5.73	**14.31**	0.26	0.33
	95% C.I.	–	0.20, 470.3	**1.31, ∞**	0.00, 2.31	0.00, 3.55
	p	–	0.473	**0.029**	0.246	0.381
Abdominal serositis	OR	1*	4.05	**11.18**	3.00	0.73
	95% C.I.	–	0.63, 35.27	**1.37, ∞**	0.44, 26.62	0.08, 5.42
	p	–	0.177	**0.022**	0.362	1.000

1 OR =  Odds ratio.

2 95% C.I.  = 95% confidence interval.

*statistically significant results (p<0.100) in bold.

**Figure 3:**
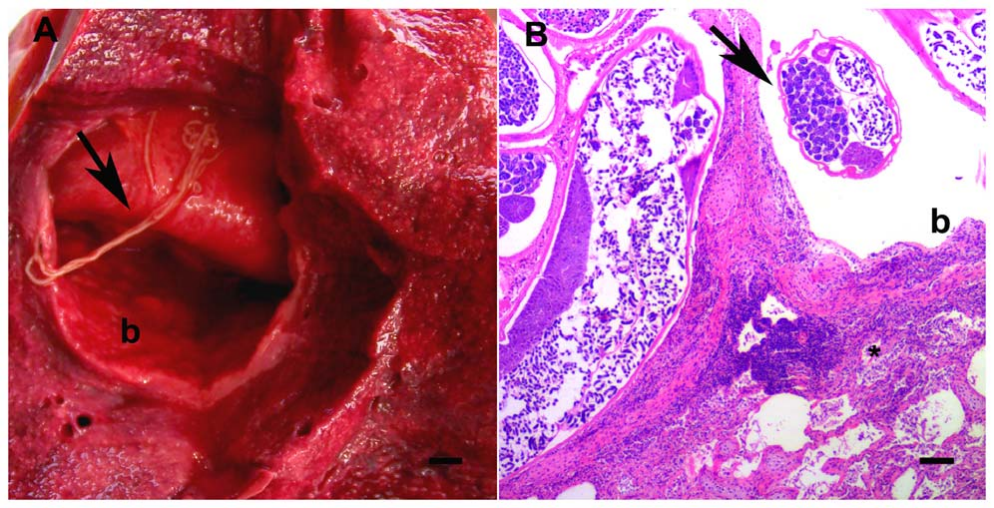
Parasitic pneumonia. A: Ectatic bronchus (b) containing thin (1–2 mm diameter), long, white helminths identified as *Halocercus* sp. (arrow). Bar  = 5 mm. B: Pulmonary helminths (arrow) in an ectatic bronchiole (b) with eosinophilic and lymphoplasmacytic interstitial pneumonia (*) and an adjacent follicle of mildly hyperplastic bronchiolar-associated lymphoid tissue (HE, bar  = 250 µm).

Mild to severe, multifocal to diffuse, acute pulmonary congestion, oedema and emphysema were common, characterized by lungs that were heavy, poorly collapsed, mottled pink to deep red and contained air-filled bullae (1–4 mm diameter) beneath the pleura and throughout the lung parenchyma. White foam filled airways of affected lungs. Variable numbers of fine white round helminths (<50×1×1 mm, *Halocercus* sp.) were present in multiple firm, white to tan, unencapsulated pulmonary nodules (<2 cm diameter) and ectatic bronchi (<8 cm diameter) in 37 animals (93%), in all ages and both sexes and species (Figure 3). Affected bronchi were lined by discontinuous attenuated epithelium, with large amounts of necrotic cellular and inflammatory debris and medium number of filarial larvae. Similar inflammation often extended into and disrupted the architecture of adjacent pulmonary parenchyma. Nematode adults, with (#5, 11, 16) or without (#6, 8, 10, 37, 40) microfilaria were present in these inflammatory lung lesions in eight (20%) animals. In addition, mild, multifocal lymphoplasmacytic and variably eosinophilic bronchointerstitial pneumonia was present in dolphins of all age classes, both sexes and species. Pneumonia was also frequently accompanied by follicular lymphoid hyperplasia of bronchus associated lymphoid tissue (18 animals; 45%).

Clustered or scattered connective tissue nodules enclosing variably mineralized necrotic debris, mixed with eosinophils, lymphocytes and plasma cells and, in some cases sections of nematodes, occurred throughout the lung parenchyma (<4 cm diameter), often close to bronchioles (16 animals, 40%). Mild to moderate, multifocal, subacute lymphoplasmacytic and variably eosinophilic tracheobronchitis, with no apparent relationship to areas of bronchiectasis or parasites, was present in 12 (44%) *T. aduncus* calves and juveniles.

Small numbers of firm, white, pleural or subpleural plaques or nodules (<5 mm diameter), occasionally containing caseous material, were seen in 16 (40%) animals. These consisted histologically of chronic pleuritis characterized by variably thick fibrous connective tissue foci containing variably mineralized necrotic inflammatory and cellular debris with moderate lymphoid follicular hyperplasia and mild pleural and interstitial fibrosis in the adjacent tissue. Mild, multifocal lymphoplasmacytic and variably eosinophilic pleuritis that was not detected on gross examination was found in 12 calves and juveniles (30%) of both species. Pleural arterioles were prominent on the visceral pleura. One male *T. aduncus* calf (#14) had a large subpleural focus of bronchiectasis (8 cm diameter) lined by compressed lung tissue (2–3 mm thick) and bronchiolar epithelium which contained a few fine filamentous white helminths (<1 mm thick, 3–5 cm long). Thick white firmly attached adhesions between the parietal and visceral pleura and the diaphragm were present in two female *T. aduncus* (#1, 23). Histologically, these consisted of bands of mature fibrous connective tissue infiltrated with small foci of lymphocytes and plasma cells. The pleural surfaces of one juvenile and one adult male *S. plumbea* (#38, 40) were covered in small fibrovascular tags (<1 cm long) with variably plasmacytic and eosinophilic pleuritis and moderate pleural and interstitial fibrosis. In *T. aduncus* no association was found between pneumonia and pleuritis (p = 0.653).

Autolysis and freezing artefact precluded detailed assessment of lymphoid tissue, however, mild to moderate follicular and paracortical lymphoid hyperplasia were seen in ten animals with respiratory tract inflammation (#6, 16, 17, 19, 22–25, 34, 36, 38) and six with lung marginal lymph node serositis characterized by aggregates of small numbers of eosinophils, lymphocytes, macrophages and plasma cells in the lung marginal lymph node connective tissue capsule (# 9, 17, 19, 22, 36, 38). Inflammation also often extended to the connective tissue between the lung and the lung marginal lymph node. Lymphoid tissue appeared depleted in two female juvenile *T aduncus* (#27, 28). Mild, focal, neutrophilic and histiocytic, necrotising lung marginal lymph node lymphadenitis was seen in association with suspected fungal hyphae in a juvenile male *T. aduncus* (#25), although the lesion was not present on serial sections stained with GMS. While 12 lung sections contained small to large numbers of mixed bacteria in blood vessels, interstitium and alveoli (H&E and Gram stains), these were not associated with necrosis or neutrophilic inflammation. A variety of bacteria were isolated on routine lung cultures, including *Pantoea agglomerans*, *Enterococcus solitarius*, *Enterobacter gergoviae*, *Shewanella algae and S. putrefaciens*, *Photobacterium damselae*, *Aeromonas media*, *Lactococcus garviae*, *Clostridium tertium*, *Streptococcus* from the *viridians* group, *Psychrobacter* sp, *Enterococcus* sp., *Micrococcus* sp., *Lactobacillus* sp., *Brevundimonas* sp., *Bacillus sp., Acinetobacter* sp. *and Proteus* sp. Lung samples from 16 animals tested by immunohistochemistry contained no dolphin morbillivirus or *Toxoplasma* antigen.

Multiple variably mineralized deposits were common, occurring beneath or replacing the bronchial and bronchiolar mucosae. Unfortunately, details of the lesions in these animals were obscured by autolysis of the bronchiolar epithelium. Both affected and unaffected dolphins originated from both regions, were from all age classes, and of both sexes and species.

**Figure 4:**
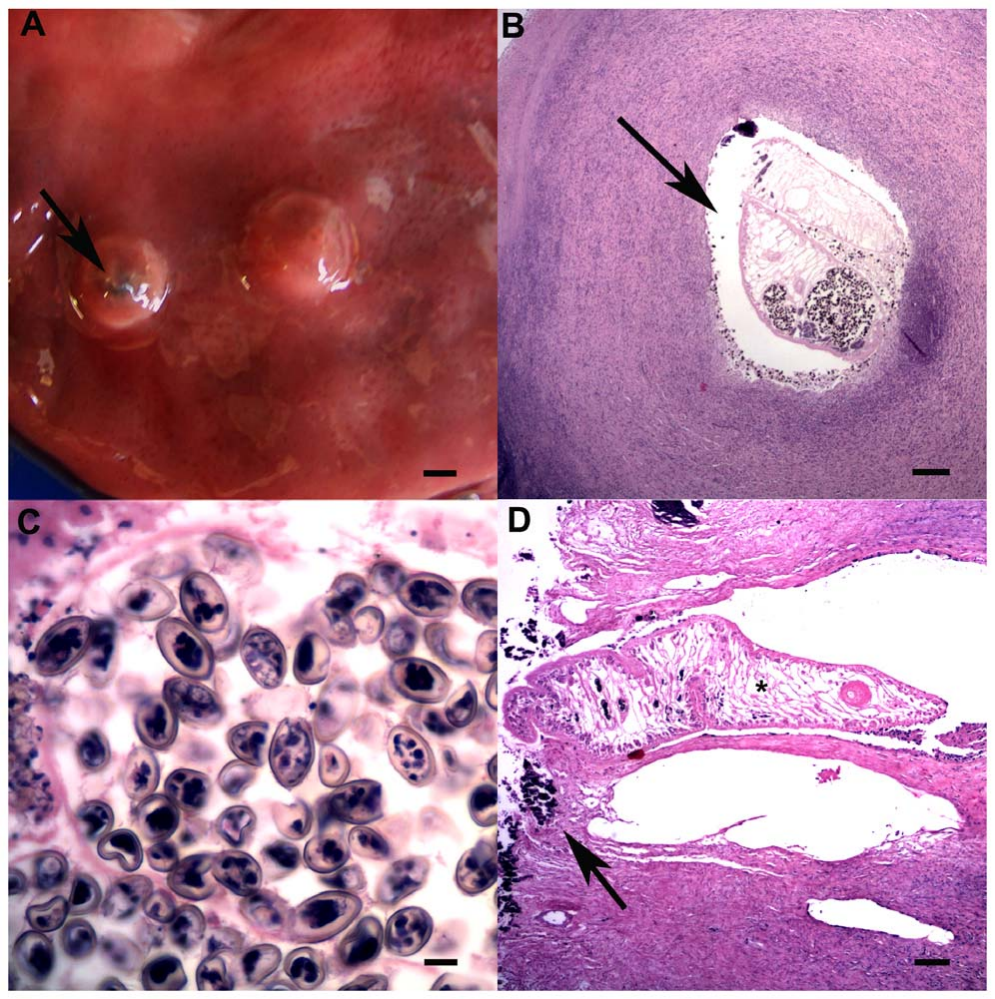
Gastric trematode associated lesions. A: Firm, round parasitic nodules (<1 cm diameter) with a small pore opening to the gastric lumen (arrow). Bar  = 0.4 cm. B: Adult trematode (arrow) in the center of a focus of extensive fibrosis (HE, bar  = 0.5 mm). C: Embryonated trematode eggs (280×160 µm, HE, bar  = 150 µm). D: Parasitic nodule with adult trematode blocking the pore and irregular mineralized foci (arrow) in the adjacent superficial gastric epithelium (HE, bar  = 500 µm).

**Figure 5:**
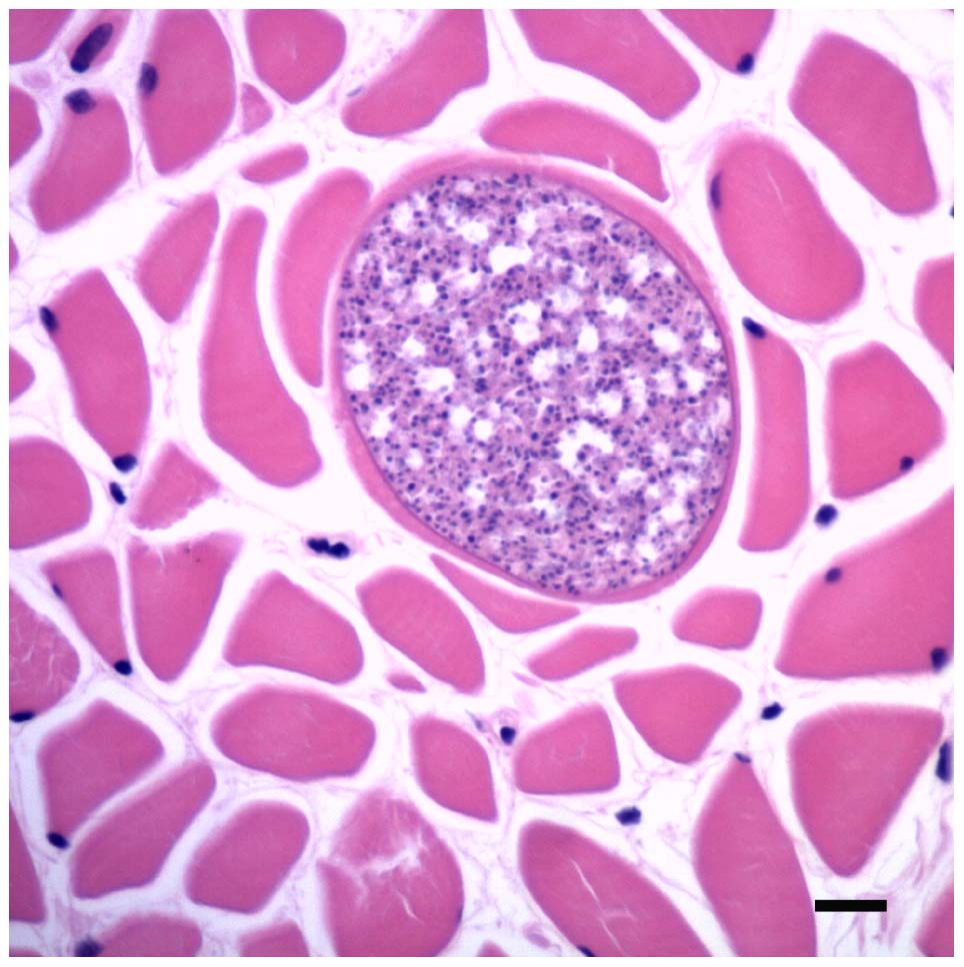
Lingual Sarcocystis. Sarcocyst containing myriad metrocytes in a muscle fiber of the tongue (HE, bar  = 150 µm).

In *T. aduncus*, all three gastric compartments contained raised, firm tan nodules with central pores (<1 cm diameter); lesions were more common in the 3^rd^ compartment (p = 0.004). Moderate to severe, multifocal, chronic lymphoplasmacytic and eosinophilic pyloric gastritis with variable calcification of the adjacent mucosa was associated with trematodes of the subfamily Brachycladiinae (Figure 4). Prevalence increased with age (p = 0.097), although this was not statistically significant in the multivariable model (p = 0.123). Eosinophilic and lymphoplasmacytic gastritis of variable severity and chronicity that was not detected on gross examination affected all three gastric compartments. The prevalence of this gastritis also increased with age (p = 0.034), as did the prevalence of similar enteritis (p = 0.002). Adult nematodes (*Anisakidae*) were found in gastro-intestinal tract of two *T. aduncus* (#26, 33). Lingual myocytes contained sarcocysts, without associated inflammation, in one *T. aduncus* calf (#13, Figure 5).

**Figure 6:**
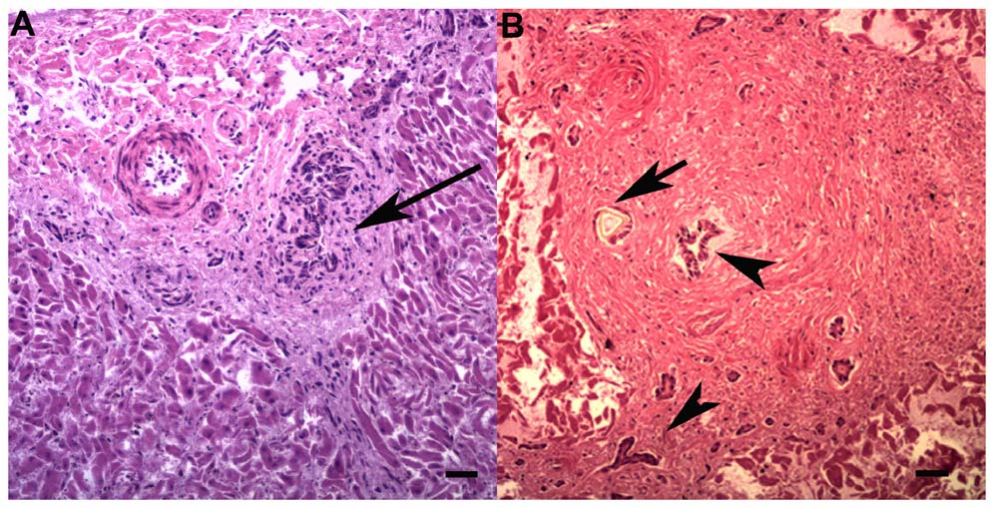
Hepatic lesions in *T. aduncus*. A: Mild proliferation (hyperplasia) of small portal bile ductules (arrow) (HE, bar  = 100 µm): B: Severe hepatic periportal fibrosis associated with a trematode egg (arrow, 100 µm diameter). Note the bile ductules with hyperplastic epithelium (arrowheads, HE, bar  = 100 µm).

Although the livers were macroscopically unremarkable, eosinophilic and variably lymphoplasmacytic, and occasionally necrotizing, periportal hepatitis and cholangitis of variable severity and chronicity were present in 21 (60%) *T. aduncus*. Adults were more often affected than calves (p = 0.044), although the association was not significant on multivariable analysis (p = 0.112). Green-brown, triangular trematode eggs (Figure 6) were found in the portal triads of three *T. aduncus* (#13, 26, 32). Moderate to marked hyperplasia of the bile duct epithelium was present in a *T. aduncus* (#21) and two *S. plumbea* (#36, 38) with cholangitis. Significantly, although two *S. plumbea* had cholangitis, no animals of this species had hepatitis (p = 0.037). Mild to severe, multifocal to diffuse increases in periportal mature fibrous connective tissue was observed with age in *T. aduncus* (p = 0.020). The presence of increased portal connective tissues was positively associated with the presence of trematode eggs (p = 0.013). Mildly to moderately increased numbers of small bile ductules in the portal triads and under the hepatic capsule were interpreted as mild to moderate bile ductular hyperplasia in 17 (42.5%) animals of all ages and both sexes. Portal connective tissue was positively associated with bile ductular hyperplasia (p = 0.009) but not with portal hepatitis (p = 0.468).

Subjectively, increased numbers of eosinophilic cell lines were present in the rib bone marrow in 22 animals of both species (75% of *T. aduncus* and 33% of *S. plumbea*) and from both regions (80% north and 63% south). Mild to moderate, multifocal, variably eosinophilic and lymphoplasmacytic oophoritis that was not detected on gross examination was found in 21% of *T. aduncus* females and one *S. plumbea* female (#37). Endometritis was more common in adults (100%) than in calves (31%) and juveniles (29%), (p = 0.044) and consisted of small clusters of lymphocytes, plasma cells and variable numbers of eosinophils and neutrophils in the endometrium. A single adult *T. aduncus* (#32) had a trematode egg associated with the endometritis. Mild to moderate, multifocal, variably eosinophilic and lymphoplasmacytic metritis that was not detected on gross examination, was found in five *T. aduncus* (#13, 30, 31, 35, 36) and a single *S. plumbea (#37)*. A positive association with age was found (p = 0.019), although this association was not significant on multivariable analysis (p = 0.107). Stamps stain for *Brucella* bacteria was negative in 12 females and all five males tested.

**Figure 7:**
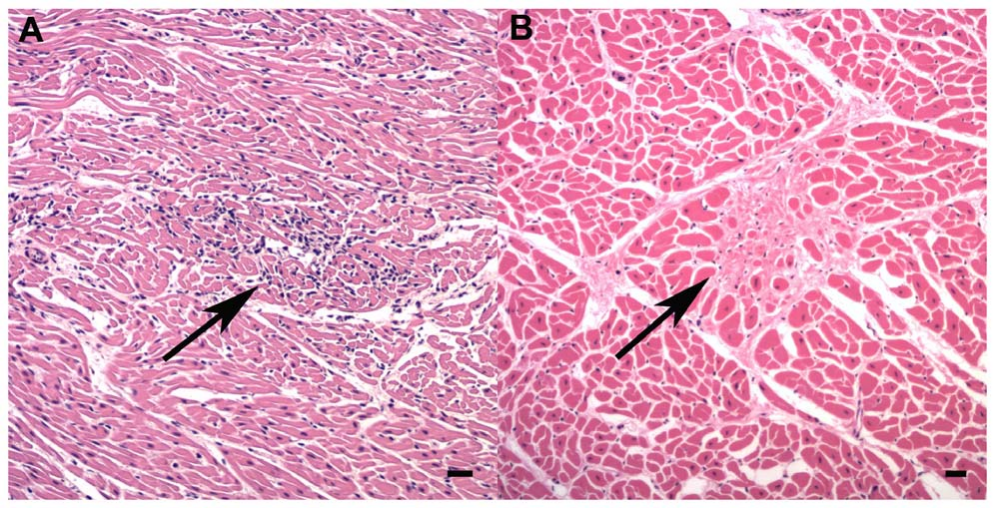
Myocardial lesions. A: Mild focal lymphoplasmacytic myocarditis (arrow) (HE, bar  = 50 µm). B: Mild focal myocardial fibrosis (arrows) (HE, bar  = 120 µm).

Mild to moderate, focal to multifocal, lymphoplasmacytic epicarditis, endocarditis and myocarditis (Figure 7A), that were not detected on gross examination, were seen in *T. aduncus* (20; 51%) but not in *S. plumbea* (p = 0.047). The highest prevalence was in juveniles (80%) (p = 0.060), although this was not significant in the multivariable model (p = 0.451). Immunohistochemistry of affected histologic sections did not demonstrate *T. gondii* antigen. Mild, focal to multifocal myocardial fibrosis (Figure 7B) was found in ten (51%) animals of both species for which heart was examined (#21, 24, 26, 29–34, 38). Prevalence increased with age (p = 0.001) and was positively associated with adrenal cortical hyperplasia (p = 0.043) but not correlated with epi-, endo-, or myocarditis (p = 0.393).

Mild, multifocal, lymphocytic meningoencephalitis was found in only seven (39%) *T. aduncus* (#7, 9, 18, 21, 25, 27, 29). Stamps and Gram histologic stains and immunohistochemistry of affected sections did not demonstrate *Brucella*, other bacteria, *T. gondii* or dolphin morbillivirus antigen.

**Figure 8:**
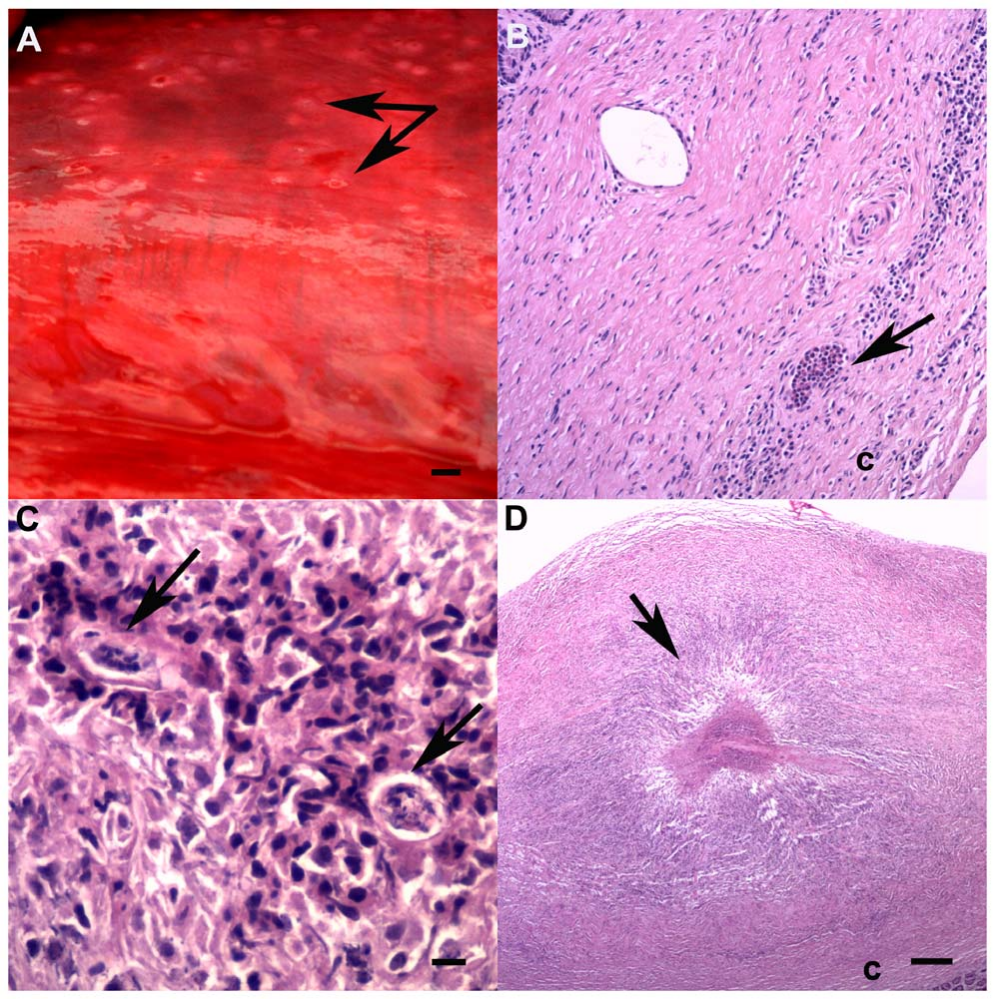
Abdominal serositis. A: Peritoneum overlying the testis contains multiple, slightly raised, firm white nodules, some of which contain depressed red centers (arrows). Bar  = 5 mm. B: Eosinophil aggregate (arrow) and lymphoplasmacytic serositis in the testicular capsule (c). Note the seminiferous tubule in the upper left corner (HE, bar  = 100 µm). C: Mesenteric lymph node serositis with intra-lesional nematode larvae in the capsule (60 µm diameter, arrows) (HE, bar  = 30 µm). D: Severe focal granulomatous testicular serositis in the testicular capsule (c) with a central area of necrosis (arrow) resembling a helminth migration tract. Note seminiferous tubules at bottom right (HE, bar  = 500 µm).

Multiple slightly raised, firm, white serosal nodules (<1 cm diameter) were present on various abdominal organs, mainly in *T. aduncus*. Animals from both regions and all age classes were affected (Figure 8). Histologically, these corresponded to mild, variably eosinophilic lymphoplasmacytic and necrotizing serositis affecting the fibrous capsule of the mesenteric lymph node (#2, 9, 13, 15, 17, 18, 20, 21, 23–26, 29, 30, 34, 35, 37, 38, 40), spleen (#9, 13, 17, 18, 23–26, 29, 32, 33, 35), liver (#4, 9, 15, 17, 21, 24, 25, 33), testis (#18, 24, 33, 38, 39), kidney (#5, 32, 36, 39), diaphragm (#7, 26, 30), and epididymis (#40), as well as adipose tissue adjacent to the mesenteric lymph node (#7, 8). Multifocal to diffuse, lymphoplasmacytic and eosinophilic inflammation was present in the mesenteric lymph node in five animals (#24, 30, 31, 34, 35), the testis in a *T. aduncus calf* (#18) and the spermatic cord in a juvenile *T. aduncus* (#38). Nematode larvae were associated with the mesenteric lymph node serositis in two juvenile male *T. aduncus* (#25, 29). These lesions were variably associated with mesenteric lymph node lymphoid hyperplasia (Table S1). The prevalence of the mesenteric lymph node serositis increased significantly with age in *T. aduncus* (p = 0.009). Male *T. aduncus* were more often affected with splenic serositis than females (p = 0.015). Renal serositis was not associated with the mild, multifocal, mainly lymphoplasmacytic, renal interstitial nephritis seen in 11 animals (Table S1).

**Figure 9:**
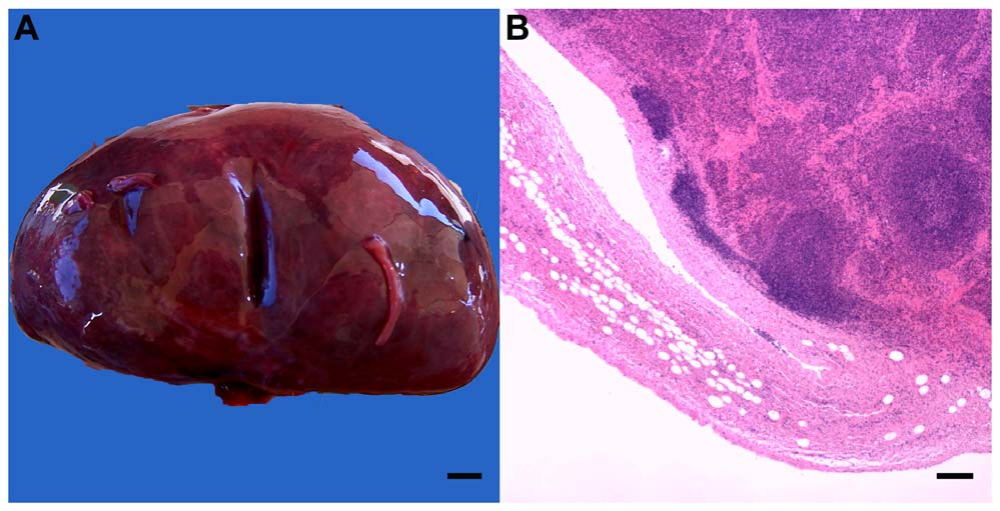
Splenic filamentous peritonitis. A: Fine long filamentous tags (1×2×30 mm) on the splenic capsule. Bar  = 10 mm. B: Splenic tag consisting of mature fibrovascular connective tissue (HE, bar  = 500 µm).

Long, slender, splenic tags occurred in a higher proportion of *T. aduncus* (49%) than *S. plumbea* (20%) (Figure 9). Histologically, these filamentous projections of the splenic capsule consisted of fibrovascular connective tissue with minimal or mild, multifocal, lymphoplasmacytic and eosinophilic inflammation. Splenic tags were significantly more common in dolphins from the southern coast (80%) than the northern coast (36%) (p = 0.027) and were associated with splenic serositis (p = 0.034).

Mild, multifocal, lymphoplasmacytic interstitial skeletal myositis was present in ten (27%) dolphins of both species and sexes from the northern region (#9, 21, 22, 23, 30, 31, 32, 33, 27, 38). Prevalence increased with age (p = 0.007). Immunohistochemistry of affected histologic sections did not demonstrate *T. gondii*. Multiple raised, pale pink cystic lesions (<1 cm diameter) containing adult *Crassicauda* sp. were associated with moderate, locally extensive, chronic, eosinophilic myositis in the musculature next to the mammary gland in one *T. aduncus* adult female (#22), which also had round basophilic crystalline structures with variable mineralized cores (interpreted as *corpora amylacea*) in the adjacent otherwise unremarkable mammary gland. Mild, multifocal, interstitial mammary gland inflammation with pleocellular infiltrates was present in two *T. aduncus* calves (#3, 6) and one juvenile (#21). Sarcocysts, without associated inflammation, were found in neck and intercostal muscle of one *T. aduncus* calf (#13).

**Figure 10:**
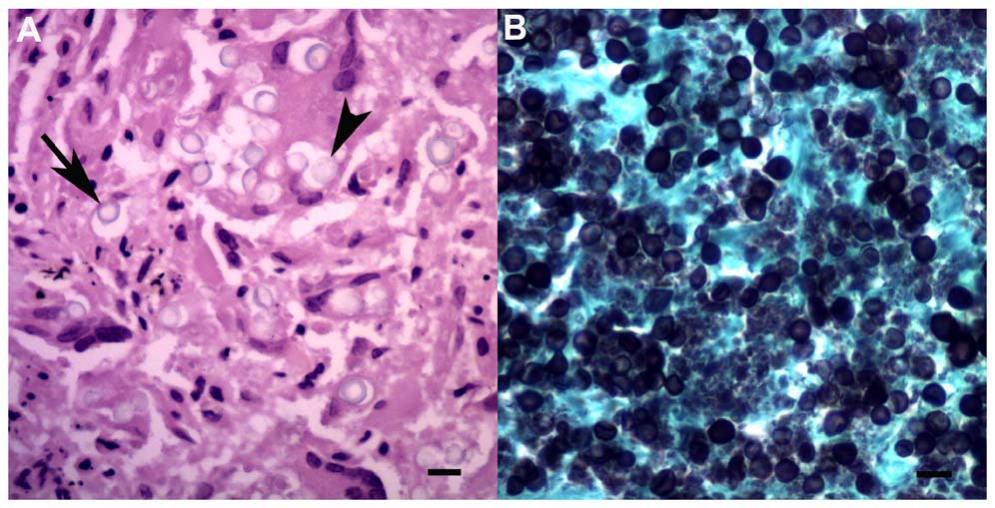
Cutaneous lobomycosis. A: Moderate numbers of round to oval refractile yeasts occur free in the subcutis (arrow) or within multinucleate giant cells (arrowhead, HE, bar  = 10 µm). B: Large numbers of deep blue-black staining yeasts (GMS, bar  = 10 µm).

All animals had superficial cutaneous linear abrasions (net marks), particularly over the thorax, flippers, flukes and head, associated with subcutaneous congestion or haemorrhage in some cases (#1, 9, 20, 30, 32). An adult male *S. plumbea* (#40) had two flat, pale-tan, lobular, cutaneous soft masses below the dorsal fin (10 mm diameter) with a light brown exudate on the cut surface. Histologically, large numbers of large foamy macrophages and rare multinucleate giant cells infiltrated the skin and subcutis with a large number of intra-lesional round yeasts (7–10 µm diameter) that stained positive on both GMS and PAS, consistent with lobomycosis (Figure 10). Small aggregates of lymphocytes, plasma cells, neutrophils or eosinophils occurred in the mammary gland interstitium of two *T. aduncus* calves (#3, 6) and one *T. aduncus juvenile (#* 8).

Mild, focal, lymphoplasmacytic and eosinophilic steatitis affecting the adipose tissue around the cervical lymph node was present in four animals (#11, 15, 20, 26). Mild, multifocal, lymphoplasmacytic and histiocytic inflammation of the capsule of the cervical lymph node and or surrounding adipose tissue was present in nine animals (#13, 17, 18, 22, 24, 26, 33, 34, 35), affecting more males (55%) than females (17%) (p = 0.045). This finding had no association with mild to moderate follicular and paracortical lymphoid hyperplasia present in this lymph node in 17 animals (Table S1).

## Discussion

This study is the first reported systematic health assessment of incidentally caught dolphins in the Southern Hemisphere. This valuable information on the current prevalence of disease in the coastal dolphin populations of South Africa can be used as a baseline for future monitoring projects.

The degree of autolysis and freezing artefact varied between animals and organs, and likely masked subtle histological features such as necrosis and tissue and inflammatory cellular detail. The presence and patterns of inflammation and parasites could, however, be confidently diagnosed, as has been documented in harbour porpoises (*Phocoena phocoena*) [38], [40], [41] and fur seals (*Arctocephalus forsteri*) [42].

Correct interpretation of tissue changes as pathological was hampered by the small sample size and the lack of standardized descriptions of tissue anatomy in dolphins. Also, in contrast to regularly dewormed domestic species, establishing normal tissue parameters is complex in free-ranging mammals which often harbour large numbers of internal parasites that may vary with age, geographical location and season. Focal (#16, 19, 25, 26, 32), multifocal (#33, 39) or diffuse (#6, 35) increases in the amounts of mature connective tissue spatially unrelated to pneumonia were compared to pulmonary connective tissue amounts in the remaining animals and subjectively diagnosed as pulmonary fibrosis. Similarly, increased amounts of periportal mature connective tissue was positively associated with age, but not with the presence of periportal hepatitis (Table S3). This may therefore be an age-related change in *T. aduncus*, although it is not clear whether this is related to trematode infections which are more numerous in older animals (Table S2). Increased numbers of small bile ducts in the portal triads and under the hepatic capsule were noted in 17 animals (Table S1); this change was subjectively associated with increased amounts of mature connective tissue, based on comparison between livers in other animals in this series and on our knowledge of similar lesions in terrestrial mammals. Too few animals were examined to assess whether the number of small bile ducts in the hepatic portal zone is variable in these species or is related to inflammatory changes. Documentation of the amount of connective tissue in well preserved tissues from new-born animals and any age-related increases, in the absence of pathological changes, would facilitate correct interpretation of the amount of pulmonary and hepatic connective tissue in these species.

Widespread tissue congestion and pulmonary emphysema and oedema are described in other net-captured cetaceans and are likely due to terminal heart failure and or drowning [37]–[39]. Clear, round vacuoles in various tissues and air emboli in blood vessels in a wide range of tissues were possibly a result of either drowning or supersaturation [84], [88]; however without more detailed studies regarding the pathophysiology of drowning in cetaceans the distinction between these two possibilities is uncertain. The histological location, absence of nuclei, and variable size of tissue and intravascular vacuoles excluded adipocytes; the absence of bacteria associated with the vacuoles (HE and Gram) make gas produced by saprophytic bacteria unlikely. However, only bubble content analysis would confirm supersaturation [89].

As expected, most of the lesions noted in these incidentally caught dolphins were mild to moderate and severe lesions were mostly focal (Table S1) and the dolphins were judged to be healthy. Blubber thickness measurements were within previously published ranges for *T. aduncus* from the KwaZulu-Natal coast [29]. No reference ranges or prior data are available for blubber thickness in *S. plumbea* from the KwaZulu-Natal coast. None of the animals with the thinnest blubber had major or multiple significant lesions and no statistical association between thinner blubber and pathology could be demonstrated. Therefore, we concluded that all animals were at least in fair nutritional condition. Although parasite levels in free-ranging animals generally have little effect on the host, factors such as stress, altered nutrition, anthropogenic factors, pollutants or concurrent disease may compromise the host's immune system and increase the severity and prevalence of parasitic infections [40]. Parasite burdens may then be used as indicators for the overall health status of an individual [40]. This assumption should, however, be made with caution, as environmental factors such as pollution may also negatively affect parasite populations [43]. Pollutant analysis on stored tissues from these dolphins would be valuable.

However, myocardial inflammation and fibrosis as well as meningoencephalitis may affect organ function and therefore be significant for the individual dolphin. Fertility and therefore population dynamics could also be affected by oophoritis, endometritis and orchitis but since one pregnant female had mild metritis, this lesion alone may not impair fertility.

Although autolysis and freezing artefact likely obscured subtle lesions, visible lesions in the respiratory and gastro-intestinal tracts were largely parasitic, as expected in incidentally caught free-ranging animals. Lesions were generally mild compared to those described in other health investigations [38], [39], [41], [44]. The presence of lungworms was less common (20%) than has been reported for stranded *T. truncatus* (77%) and *S. coeruleoalba* (76.5%) from the Northern Hemisphere [45], [46]. Eosinophilic pneumonia, even in the absence of visible parasites, was likely parasitic [47], [48].

*Halocercus* spp. are common in the lungs of many dolphin species, although the complete life cycle remains unknown [44], [49]. They are generally considered to be of no clinical importance in *T. truncatus* from Florida [45]. Since parasites were recovered more often from calves than from juveniles, and no parasites recovered from adults, the infestation is likely established *in utero* or through milk ingestion [45], [49]. Adult animals more often showed only chronic or resolving infections; however, heavily infested adults that died due to parasitism would have been missed in this survey. The variable lymphoplasmacytic inflammation and accompanying follicular lymphoid hyperplasia may indicate the presence of persistent foreign antigen and activation of the adaptive immune response despite clearance of the infestation in older animals [50]. As has been described previously [45], pulmonary interstitial fibrosis was significantly more common in older animals. Interstitial pulmonary fibrosis is a sequel to repetitive, persistent, or severe damage to the endothelial or epithelial cells, inflammation of the alveolar septa, or chronic pulmonary hypertension [51]. In dolphins it has commonly been reported in chronic morbillivirus [52]–[54] and parasitic infections [44], [45]. However, no association between fibrosis and pneumonia or pulmonary verminosis could be demonstrated in this study.

Gastric parasitic nodules due to the trematode *Pholeter gastrophilus* infestation are a common incidental finding in dolphins [49], [55], [56]. As described previously, nodules were mainly in the pyloric compartment. Nematodes belonging to the family Anisakidae have an indirect life cycle, with animals ingesting infective larvae in infected fish and squid [49]. This likely explains the higher prevalence in juveniles and adults, since calves only become infected once they start consuming fish. Observed species differences in the prevalence of parasitic lesions in the liver, stomach, spleen, lung and lymph nodes may be a result of the small sample size of *S. plumbea*. Alternatively, the parasites that cause these lesions could be host specific due to consumption of different fish and squid species that act as intermediate or paratenic hosts [49]. Of the 94 prey species recorded in *T. aduncus* and 54 prey species in *S. plumbea*, only 25 species are eaten by both *T. aduncus* and *S. plumbea* [57], [58]. Changing diet due to changes in prey population dynamics, climate change and or anthropogenic influences may affect parasite loads and is a key topic for future research.

Parasites, including the trematodes *Campula*, *Oschmarinella*, and *Brachycladium* (formerly *Zalophotrema*) which have been found in hepatic ducts, were the most likely cause of the hepatitis and periportal hepatitis in *T. aduncus* [44], [49], [56]. The life cycle of these brachycladiids is not known [49]. The eosinophilic öophoritis, endometritis, metritis and orchitis were also probably caused by parasites, supported by the trematode egg present in one case. The positive association with age (up to 100% of adult animals) suggests an indirect life cycle. Small sample size, bias towards younger animals and autolysis precludes a definitive diagnosis of increased bone marrow eosinophilic myelopoiesis; however, a predominance of eosinophilic bone marrow cell lines could reflect the widespread parasitism in these dolphins. Sarcocysts have not previously been reported in dolphins from South African waters, although they have been reported in other cetacean populations [18], [49], [59]–[62]

Widespread serosal eosinophilic or fibrotic abdominal serosal lesions were reported to have increased in prevalence in 2009 (*pers. comm*. S. Plön). Similar lesions are described in in domestic horses with *Strongylus* spp migrations, and in domestic pigs due to chronic bacterial serositis. Most of the lesions were chronic with no definitive indication of aetiology. However, parasite larvae were found in the capsules of two mesenteric lymph nodes, and a necrotic tract suggestive of a migration tract was found in another mesenteric lymph node. Lack of association between serosal lesions and pulmonary verminosis, hepatic trematode eggs, or gastric trematodes may be due to the fact that these parasites were not the cause of the lesions, or perhaps due to temporal changes in lesion location and severity over the life cycle of the parasite. Changes in the ecology of food species acting as parasite intermediate hosts could explain the apparent changes in the prevalence of these lesions. Further research is needed on the identity of the parasite, its life cycle and the possible changes in host, environment and prey factors that may influence parasitic loads. Although the inflammatory nature of the splenic serositis resembles that in other abdominal organs, the aetiology of the splenic tags remains uncertain and further research is needed to determine their significance and explain why they are more common in *T. aduncus*, particularly from the southern region.

No histological or immunohistochemical evidence of dolphin morbillivirus infection, brucellosis or toxoplasmosis was found. However, cetacean morbillivirus antibodies were previously found in a *D. delphinus* that stranded approximately 350 km south of the study area [65]. Regrettably, no pathological information is available for this animal and paired serum samples could not be taken to confirm active infection. This population of dolphins may be less susceptible to these diseases than other populations. Alternately, the prevalence of these diseases may have been too low to detect in our study. However, the absence of histological or immunohistochemically stained antigen in the tissues from these dolphins may also have occurred due to poor tissue preservation or loss of antigen integrity due to formalin fixation. The antibody used to detect morbillivirus antigen was a pan-morbillivirus antibody and has been used with success in *Phoca vitulina* (harbour seal) [33], and *S. coeruleoalba* [38]. Commercially available immunohistochemical stains used in this study have been used effectively to detect *T. gondii* in dolphins [66]. The modified ZN (Stamps) stain is an accepted method of demonstrating *Brucella* spp. organisms in tissues [67], [68]. This is a crucial area for future research, given the presence of inflammatory lesions compatible with these diseases and their worldwide distribution. Continued monitoring of these dolphin populations is needed as reliable detection of infectious agents present at low prevalence can only be accomplished by testing larger numbers of animals but access to live free-ranging coastal dolphins is limited [65], [69]. Microbiological culture and biotyping of brain, spleen and reproductive tract isolates will be conducted in future. Serological and molecular diagnostic tests for *Brucella* spp. and *T. gondii* are also needed. If these dolphin populations are in fact naïve to these pathogens, their introduction could have devastating consequences, as has been documented previously in other populations elsewhere during morbillivirus epidemics [5], [52], [54], [70]–[73].

No animals had lesions consistent with bacterial pneumonia and no primary bacterial pathogens were isolated from the lung. However, autolysis and freezing may have compromised culture success. Isolation of opportunistic bacteria such as *Aeromonas media* and *Photobacterium damselae* is consistent with previous reports [72]. *Shewanella algae* is commonly isolated from marine environments, and is an opportunistic human pathogen [74]. Remaining bacteria were considered contaminants or normal commensals.

Granulomatous dermatitis associated with fungi is consistent with the zoonotic disease lobomycosis [5], [75], [76]. This is, to our knowledge, the first confirmed report of lobomycosis in South African waters, although macroscopic lobomycosis-like disease has been documented in other Indian Ocean populations of *T. aduncus* [77]. Impaired adaptive immunity was found in endemically affected *T. truncatus* from the Indian River Lagoon, Florida [78]. The exact aetiology of the immunosuppression in dolphins has not yet been determined, but both environmental contaminants, such as mercury and polychlorinated biphenyls, and chronic stress as result of anthropogenic factors have been suggested [76], [78]. No evidence of immunosuppression was found histologically in the dolphins in this study, although differential white cell counts, determination of lymphocyte subpopulations, phagocytic activity and lysozyme activity, amongst other tests [78], were not possible in incidentally caught animals.

While some variation in the width of the adrenal cortex and occasional cortical nodules were seen in the cortex or medulla in these animals, such variation could have been due to differing planes of section. Blood and faecal adrenocortical hormone assays, adrenal weights and objective measurement of adrenal cortico-medullary ratios by point-counting techniques [32] as well as systematic evaluation of the pituitary are needed to evaluate the possibility of stress in this dolphin population. Adrenal hyperplasia has been attributed to chronic stress from long-term debilitating disease or injury in *T. truncatus* in the Gulf of Mexico [32], [79]; however, the animals in this study had relatively mild pathology. Environmental stressors, such as competition for resources, and anthropogenic factors, such as boat traffic, seismic or military activities warrant evaluation. Myocardial fibrosis is a non-specific indication of prior tissue damage due to inflammation or necrosis. Myocardial necrosis and fibrosis in stranded and incidentally caught *T. truncatus* and *S. coeruleoalba* from the Gulf of Mexico were attributed to the acute and chronic effects, respectively, of high catecholamine levels [79]. The association of cardiac fibrosis with age may indicate that the effects are cumulative. Cardiac fibrosis was not associated with myocarditis in *T. aduncus*; however, the small sample size precludes definitive conclusions on the aetiology of either lesion. Similarly, the small sample size, including only one adult *S plumbea*, may account for the absence of epicarditis, endocarditis or myocarditis seen in this species. Although mild cutaneous depigmentation (#1), lacerations (#6, 26, 31), and barnacles (#3) were documented, inter and intra-specific aggression could not be reliably distinguished from boat strike or other anthropogenic injury.

The higher numbers of *T. aduncus*, caught in the nets all along the coast likely reflects the relative population size and more widespread distribution of this species [21], [22]. All five *S. plumbea* were caught on two adjacent beaches in the northern region (Figure 1), where they occur in higher numbers than in the south [80], [81]. The fact that calves and juveniles are more inquisitive and inexperienced may explain why *T. aduncus* calves and juveniles were caught more often than adults [82]. Females with calves also feed closer to shore, and therefore to the nets, which results in higher capture rates of adult females and calves [64], [83].

Mineralization of the bronchiolar epithelium has previously been attributed to lungworm infection [85], [86]. Bronchiolar mineralization is not a common feature of verminous pneumonia in cetaceans [38], [44], but is occasionally seen in harbour porpoises from the North Sea (P. Wohlsein, *pers. comm*.). Foreign particles are thought to accumulate in the lung due to the inability of dolphins to cough. These particles become inspissated, undergo calcification and are later incorporated into the bronchial wall [85]. Additional investigations are underway to determine the distribution and exact location of the material. Small foci of mineralisation were present in 24 dolphins in a wide range of tissues, in addition to the airways (Table S1). In mammals, metastatic tissue mineralisation due to disturbed calcium and phosphorus metabolism typically occurs on the intercostal pleura, pulmonary and renal cortical basement membrane, and the middle and deep gastric mucosa [90]. Since these sites were not involved, and no indication of renal failure, neoplasia, or granulomatous inflammation that could result in secondary hyperparathyroidism were present, the mineralisation seen in these dolphins was assumed to be dystrophic changes due to minor tissue damage. However, since neither pituitary nor parathyroid glands were routinely sampled we cannot rule out the possibility of altered calcium homeostasis in these dolphins. We consider that nutritional hyperparathyroidism (due to altered calcium, phosphate or Vitamin D metabolism) is unlikely to be common in free-ranging animals; and cannot rule out the possibility of emerging secondary marine plant intoxication through ingestion of herbivorous fish.

## Conclusion

In the first systematic health assessment of incidentally caught coastal dolphins in the Southern Hemisphere, we report the first confirmed cases of lobomycosis and sarcocystosis in dolphins from the South African coast. While optimum samples are not provided by frozen, incidentally caught animals, this study still yielded valuable information on the current prevalence of disease in the two dolphin populations, which can be used as a baseline for future monitoring projects, not only of the health status of the population, but also that of the environment. This may prove particularly important for *S. plumbea*, whose coastal habitat, restricted distribution range, and small population size make it prone to a number of threats, including anthropogenic impacts. These findings further highlight the importance of disease investigation in marine mammals.

## Supporting Information

Table S1 **Summary of mild, moderate and severe lesions and overall health status for each of 35 Indian Ocean bottlenose (T. aduncus) and five Indo-Pacific humpback (S. plumbea) dolphins incidentally caught in shark nets along the KwaZulu-Natal coast, South Africa, 2010-2012.**
(DOCX)

Table S2 **Complete pathological findings for indicating occurrence (lesion/number of organ evaluated) and percentage per species, age group, and region (for both species combined).**
(DOCX)

Table S3 **Common pathology observed in Tursiops aduncus and associations with sex, age and region.**
(DOCX)
