# Supplementary material for: A Systematic Health Assessment of Indian Ocean Bottlenose (Tursiops aduncus) and Indo-Pacific Humpback (Sousa plumbea) Dolphins Incidentally Caught in Shark Nets off the KwaZulu-Natal Coast, South Africa
Source: PLoS One. 2014 Sep 9;9(9):e107038. doi: 10.1371/journal.pone.0107038 (PMC4159300; doi:10.1371/journal.pone.0107038)
Supplement: Table S1 — Summary of mild, moderate and severe lesions and overall health status for each of 35 Indian Ocean bottlenose (T. aduncus) and five Indo-Pacific humpback (S. plumbea) dolphins incidentally caught in shark nets along the KwaZulu-Natal coast, South Africa, 2010-2012. (DOCX) [file pone.0107038.s001.docx]

Table S1: Summary of mild, moderate and severe lesions and overall health status for each of 35 Indian Ocean bottlenose (*T. aduncus*) and five Indo-Pacific humpback (*S. plumbea*) dolphins incidentally caught in shark nets along the KwaZulu-Natal coast, South Africa, 2010-2012.

| ID+ | Spp# | Sex^ | Age * | Region^##^ | Lesion severity | | | Health status |  |
| --- | --- | --- | --- | --- | --- | --- | --- | --- | --- |
|  |  |  |  |  | Mild | Moderate | Severe |  | |
| 1  (N3573) | Ta | F | C | N | Multifocal cutaneous depigmentation (medial fins); cervical lymph node sinusoidal eosinophilia and lymphoid hyperplasia; filamentous helminths in sinus behind left eye, in the goosebeak and oesophagus (1 - 2 cm); multifocal bronchiolar mineralisation; multifocal eosinophilic interstitial pneumonia; acute pulmonary oedema. | Focal acute subcutaneous haemorrhage; multifocal bronchiole-associated follicular lymphoid hyperplasia; pleural adhesions; acute tissue congestion; splenic capsular petechiae and ecchymoses. | Multifocal pulmonary emphysema. | Pleural adhesions could affect diving and feeding success after weaning. | |
| 2 (N3576) | Ta | F | C | N | Focal epidermal hyperplasia; focal perithyroid lymphocytic steatitis; acute focal thymic haemorrhage; multifocal lymphoplasmacytic interstitial pneumonia (with perivascular pleuritis, arteritis, interstitial fibrosis), segmental epicardial phlebitis, steatitis, myocarditis, periportal hepatitis, metritis; multifocal lymphoplasmacytic, eosinophilic capsular mesenteric lymphadenitis; renal corticomedullary lymphoid hyperplasia. | Cervical lymph node lymphoid hyperplasia; acute pulmonary congestion, oedema, emphysema; multifocal bronchiolar mineralization; intravascular eosinophilic leucostasis; acute tissue congestion; multifocal periportal fibrosis, bile duct, ductular hyperplasia; bone marrow eosinophilic myelopoiesis. |  | Healthy. | |
| 3 (N4342) | Ta | F | C | N | Focal epidermal hyperplasia, lymphoplasmacytic subcutaneous steatitis (*Xenobalanus globicipitis*); multifocal lymphoplasmacytic mastitis, enteritis; multifocal bronchiolar mineralisation; acute pulmonary oedema; focal lymphocytic gastritis; acute tissue congestion; splenic eosinophilic intravascular leucostasis; diffuse lymphoplasmacytic, neutrophilic endometritis. | Focal subpleural emphysema; diffuse alveolar emphysema; multifocal small intestinal mucosal lymphoid hyperplasia; mesenteric lymph node lymphangiectasia; multifocal lymphoplasmacytic, eosinophilic periportal hepatitis; perisplenic fat necrosis. |  | Healthy. | |
| 4 (N4346) | Ta | F | C | N | Cervical lymph node lymphoid hyperplasia; multifocal lymphoplasmacytic, neutrophilic, histiocytic interstitial pneumonia; multifocal bronchiolar mineralisation; focal lymphoplasmacytic, histiocytic gastritis; acute tissue congestion; diffuse eosinophilic enteritis; bile ductular hyperplasia (portal triads, hepatic capsule); splenic fibrovascular tags. | Acute alveolar emphysema; diffuse pulmonary alveolar histiocytosis; lymphoplasmacytic, eosinophilic necrotizing periportal parenchymal, capsular hepatitis. | Splenic lymphoid hyperplasia. | Healthy. | |
| 5 (N4347) | Ta | F | C | N | Multifocal lymphoplasmacytic, eosinophilic verminous bronchointerstitial pneumonia; multifocal lymphocytic endocarditis, capsular nephritis; focal renal dysplasia. | Acute pulmonary oedema; acute alveolar emphysema; multifocal bronchiolar mineralisation; acute tissue congestion bone marrow eosinophilic myelopoiesis. | Single white firm nodule (0.5 cm diameter) in left lung consisting of necrotizing verminous pneumonia (microfilaria). | Healthy. | |
| 6 (N4531) | Ta | F | C | N | Cutaneous puncture wound over left mammary gland; multifocal lymphocytic, neutrophilic mastitis, ductular epithelial hyperplasia; eosinophilic intravascular leucostasis; multifocal bronchiolar, mammary, renal medullary mineralisation; multifocal lymphoplasmacytic pharyngitis, oesophagitis, enteritis, pancreatitis, pituitary adenitis, periportal hepatitis; acute pulmonary oedema, congestion; lung marginal lymph node sinusoidal eosinophilic leucostasis; multifocal neutrophilic, lymphoplasmacytic necrotising myocarditis, endocarditis; diffuse plasmacytic, eosinophilic pleuritis; diffuse eosinophilic enteritis; multifocal lymphoplasmacytic, histiocytic, eosinophilic necrotising hepatitis, adrenal adenitis; focal histiocytic, neutrophilic necrotising subcapsular interstitial nephritis; focal acute adrenal capsular haemorrhage; multifocal lymphocytic, eosinophilic oophoritis. | Bronchiole-associated, lung marginal lymph node lymphoid hyperplasia; focal acute mucopurulent bronchopneumonia; acute pulmonary emphysema; small firm white nodules of lymphocytic, eosinophilic interstitial pneumonia with extensive fibrosis; focal lymphoplasmacytic pleuritis with marked fibrosis; multifocal lymphoplasmacytic , eosinophilic gastritis; focal gastric, small intestinal mucosal lymphoid hyperplasia; acute tissue congestion. | Diffuse chronic fibrous pleuritis; multifocal to coalescing lymphoplasmacytic, eosinophilic verminous bronchopneumonia, mild bronchiectasis, fibrosis; splenic lymphoid hyperplasia; bone marrow eosinophilic myelopoiesis. | Myocarditis and endocarditis could affect diving and feeding success; oophoritis could affect breeding success if it persists to maturity. | |
| 7 (N4532) | Ta | F | C | N | Multifocal lymphoplasmacytic pharyngitis, salivary adenitis, tracheitis, gastritis, diaphragmatic serositis, steatitis (adjacent to mesenteric lymph node), pituitary adenitis, meningitis; diffuse acute eosinophilic interstitial pneumonia; multifocal neutrophilic, lymphoplasmacytic, eosinophilic interstitial pneumonia; multifocal bronchiolar, gastric submucosal mineralisation; diffuse acute pulmonary emphysema; multifocal lymphoplasmacytic, eosinophilic pleuritis, perivascular (aortic) arteritis, subserosal enteritis; multifocal lymphoplasmacytic necrotizing endo, myocarditis; umbilical artery subintimal mineralisation, haematoidin; multifocal, perivascular lymphoplasmacytic, neutrophilic encephalitis with swollen axons. | Acute tissue congestion; eosinophilic intravascular leucostasis; diffuse pulmonary emphysema; multifocal splenic, mesenteric lymph node, gastric, small intestinal associated lymphoid hyperplasia; multifocal lymphocytic glossitis with mild muscle necrosis; lymphoplasmacytic periportal hepatitis. | Acute pulmonary oedema. | Myocarditis could affect diving and feeding success; necrotizing glossitis could affect feeding success. | |
| 8 (N4536) | Ta | F | C | N | Eosinophilic intravascular leucostasis; mild alveolar histiocytosis; multifocal lymphoplasmacytic, eosinophilic verminous bronchointerstitial pneumonia with moderate fibrosis (*Halocercus* spp); lung marginal lymph node multifocal eosinophilic capsular lymphadenitis; multifocal lymphoplasmacytic epi, myocarditis; multifocal lymphoplasmacytic, eosinophilic enteritis; focal lymphoplasmacytic perivascular steatitis (adjacent to mesenteric lymph node), mesenteric lymph node arteritis; acute peripancreatic fat necrosis; multifocal renal interstitial mineralisation; splenic extramedullary haematopoiesis; multifocal lymphoplasmacytic perivascular oophoritis; focal eosinophilic necrotising oophoritis; segmental mucometra; multifocal eosinophilic superficial endometritis; focal lymphocytic scleritis. | Cervical lymph node medullary sinus histiocytosis; diffuse lymphoplasmacytic tracheitis; acute tissue congestion; multifocal bronchiolar-associated lymphoid hyperplasia; lung marginal lymph node intravascular eosinophilic, neutrophilic leucostasis; focal lymphocytic, eosinophilic perivascular subserosal enteritis; mesenteric lymph node lymphangiectasia; lymphoplasmacytic, neutrophilic, eosinophilic necrotizing periportal hepatitis. | Acute pulmonary oedema; ovarian rete tubule ectasia. | Myocarditis could affect diving and feeding success; oophoritis and mucometra could affect reproductive success if it persists to maturity. | |
| 9 (N4639) | Ta | F | C | N | Diffuse subcutaneous acute congestion, haemorrhage ventral chest, abdomen; multifocal lymphoplasmacytic panniculitis, intercostal myositis, pharyngitis, oesophagitis, periportal hepatitis, pancreatitis, interstitial cortical, medullary nephritis, endometritis, scleritis, perivascular adrenal adenitis, meningoencephalitis, trigeminal neuritis; multifocal lymphoplasmacytic, histiocytic myositis, capsular hepatitis; eosinophilic intravascular leucostasis; multiple white firm nodules of pulmonary fibrosis, necrosis; lung marginal lymph node multifocal lymphoplasmacytic capsular lymphadenitis, sinusoidal eosinophilia; acute tissue congestion; multifocal lymphoplasmacytic, eosinophilic transmural gastroenteritis, transmural cystitis, trigeminal perineuritis, endo, myocarditis; multifocal gastric mucosal hyperplasia; multifocal gastric mucosal squamous metaplasia; lung marginal lymph node, mesenteric lymph node lymphoid hyperplasia; periportal bile ductular hyperplasia; renal corticomedullary lymphoid hyperplasia; multifocal lymphoplasmacytic, histiocytic, eosinophilic perirenal steatitis; splenic fibrovascular tag; splenic lymphoid hyperplasia; marked acute cranial spinal cord haemorrhage. | Mesenteric lymph node lymphangiectasia; diffuse lymphoplasmacytic, eosinophilic capsular mesenteric lymphadenitis (with sinusoidal histiocytosis, eosinophilia), perivascular peripancreatic steatitis, capsular splenitis; bone marrow eosinophilic myelopoiesis. | Multifocal lymphoplasmacytic, eosinophilic bronchointerstitial pneumonia. | Suckling; widespread inflammation could affect growth rate, endometritis could affect reproductive success if it persists to maturity. | |
| 10 (N4343) | Ta | F | C | S | Firm white nodules of lymphoplasmacytic, eosinophilic verminous bronchopneumonia with marked fibrosis, bronchiectasis; acute tissue congestion; acute pulmonary emphysema; multifocal bronchiolar mineralisation; focal renal cortical fibrosis; multifocal fat necrosis (adjacent to adrenal, kidney, ovary, bladder). | Diffuse acute pulmonary oedema; mineralised foci in renal tubules. |  | Healthy. | |
| 11 (N4344) | Ta | F | C | S | Focal lymphocytic, eosinophilic steatitis (around cervical lymph node); acute tissue congestion lymphocytic periportal hepatitis; haematoidin in the umbilical artery; splenic extra-medullary haematopoiesis; inactive bone marrow. | Pulmonary oedema, emphysema; splenic fibrovascular tags. | Diffuse lymphoplasmacytic, histiocytic hyperplastic bronchitis; small ( < 1cm) white foci of lymphoplasmacytic, eosinophilic bronchopneumonia with moderate bronchiectasis large numbers of *Halocercus* spp., moderate lymphoid hyperplasia; multifocal necrotising interstitial pneumonia (with large numbers of free microfilaria). | Healthy, suckling. | |
| 12 (N4539) | Ta | F | C | S | Subcutaneous white firm nodular focus of lymphoplasmacytic necrotizing verminous cellulitis caudal to dorsal fin; multifocal lymphocytic cervical lymph node arteritis; multifocal bronchiolar, renal tubular luminal mineralisation; focal bronchiolar-associated lymphoid hyperplasia; focal lymphoplasmacytic myocarditis; focal neutrophilic, lymphocytic, histiocytic necrotising periportal hepatitis; splenic fibrovascular tags; eosinophilic myelopoiesis. | Acute pulmonary emphysema, congestion, haemorrhage; multifocal lymphoplasmacytic, histiocytic, eosinophilic bronchointerstitial pneumonia; acute tissue congestion; focal chronic lymphoplasmacytic, eosinophilic necrotizing gastritis (with trematode eggs). | Eosinophilic intravascular leucostasis; acute dural, synovial cranial cervical haemorrhage. | Healthy. | |
| 13 (N4642) | Ta | F | C | S | Intravascular eosinophilic leucostasis; focal lymphocytic capsular thyroiditis; focal lymphoplasmacytic, eosinophilic perithymic steatitis; multifocal plasmacytic, eosinophilic tracheitis; focal tracheal, bronchiole associated lymphoid hyperplasia; acute pulmonary oedema; diffuse eosinophilic interstitial pneumonia; multifocal lymphoplasmacytic pleuritis (with interstitial fibrosis), cystitis, transmural metritis; acute tissue congestion; multifocal lymphoplasmacytic endo, myocarditis; multifocal gastric, gastric lymph node, renal corticomedullary lymphoid hyperplasia; neck, lingual, intercostal muscle sarcocytosis; diffuse eosinophilic necrotizing mesenteric lymphadenitis; multifocal lymphoplasmacytic, eosinophilic mesenteric lymph node capsular lymphadenitis, periportal hepatitis (with a trematode egg, fibrosis), capsular splenitis. | Cervical, mesenteric lymph node, splenic lymphoid hyperplasia; acute pulmonary congestion, emphysema; multifocal lymphoplasmacytic, eosinophilic cervical capsular lymphadenitis, interstitial pneumonia, gastritis, enteritis; multifocal bronchiolar mineralisation; chronic parasitic gastritis (with adult trematode, trematode eggs); moderate multifocal gastric mucosal lymphoid hyperplasia; focal chronic eosinophilic, lymphoplasmacytic necrotizing parasitic gastritis ( with trematode eggs, multifocal gastric mucosal mineralisation); bile ductular hyperplasia. |  | Myocarditis could affect diving and feeding success; metritis could affect reproductive success. | |
| 14 (N4345) | Ta | M | C | N | Multifocal thyroid cysts; acute tissue congestion; focal lymphocytic, eosinophilic cholangitis; bone marrow eosinophilic myelopoiesis. | Diffuse lymphoplasmacytic, eosinophilic tracheobronchitis; pulmonary oedema; multifocal bronchiolar mineralisation; multifocal lymphocytic, neutrophilic, eosinophilic interstitial pneumonia with multifocal lymphoid hyperplasia; focal lymphocytic, eosinophilic perivascular pleuritis. | Subpleural focal bronchiectasis (right dorsal lung) containing small number of white nematodes (<1mm thick, 3 - 5 cm long); subintimal mineralisation (umbilical artery). | Healthy. | |
| 15 (N4348) | Ta | M | C | N | Multifocal lymphoplasmacytic pharyngitis, bronchitis, pleuritis; epicarditis, myocarditis, glossitis, capsular hepatitis, interstitial nephritis, perirenal steatitis, adrenal adenitis, ductal sialoadenitis; focal plasmacytic steatitis (adjacent to cervical lymph node); acute pulmonary oedema, emphysema; multifocal cervical lymph node, bronchiolar mineralisation; multifocal oesophageal, gastric, small intestinal, renal corticomedullary junction lymphoid hyperplasia; mesenteric lymph node lymphangiectasia; diffuse eosinophilic periportal hepatitis; acute perisplenic fat necrosis. | Multifocal lymphoplasmacytic, neutrophilic, eosinophilic bronchopneumonia; acute tissue congestion; focal chronic lymphoplasmacytic gastritis; multifocal to diffuse neutrophilic capsular mesenteric lymphadenitis. | Cervical lymph node lymphangiectasia. | Healthy, neonate. | |
| 16 (N4349) | Ta | M | C | N | Acute pulmonary emphysema; multifocal bronchiolar, mesenteric lymph node mineralisation; marginal lymph node lymphoid hyperplasia; lymphoplasmacytic, eosinophilic necrotising periportal hepatitis; haematoidin (umbilical artery); splenic extramedullary haematopoiesis; intravascular eosinophilic leucostasis; focal lymphoplasmacytic, histiocytic, neutrophilic balanitis. | Acute tissue congestion; multifocal lymphoplasmacytic, eosinophilic, histiocytic necrotising verminous bronchointerstitial pneumonia (microfilaria). | Diffuse lymphoplasmacytic, histiocytic tracheitis; acute pulmonary oedema; white firm subpleural foci of pulmonary fibrosis, necrosis with lymphoid hyperplasia. | Balanitis could affect reproductive success. | |
| 17 (N4640) | Ta | M | C | N | Multifocal lymphoplasmacytic cervical lymph node capsular lymphadenitis with mild lymphoid hyperplasia; multifocal bronchiolar mineralisation; acute tissue congestion; multiple red raised foci of eosinophilic lung marginal lymph node capsular lymphadenitis, capsular splenitis, hepatitis; focal lymphocytic, eosinophilic perivascular myocarditis, capsular mesenteric lymphadenitis, enteritis, splenitis; multifocal lymphocytic gastritis, perirenal steatitis; focal lymphocytic optic perineuritis. | Acute pulmonary oedema, emphysema; lung marginal, mesenteric lymph node lymphoid hyperplasia; acute tissue congestion; multifocal gastric mucosal lymphoid hyperplasia; periportal bile ductular hyperplasia; bone marrow eosinophilic myelopoiesis. | Focal lymphoplasmacytic, eosinophilic bronchointerstitial pneumonia; focal chronic lymphoplasmacytic, eosinophilic parasitic gastritis (with an adult trematode, eggs ). | Healthy. | |
| 18 (N4641) | Ta | M | C | N | Multifocal lymphoplasmacytic cervical lymph node capsular lymphadenitis, capsular splenitis, superficial balanitis; multifocal bronchiolar, gastric mucosal, renal tubular luminal mineralisation; acute pulmonary oedema; multifocal eosinophilic pleuritis; acute tissue congestion; multifocal gastric mucosal lymphoid hyperplasia; multifocal eosinophilic, lymphoplasmacytic gastritis, subserosal enteritis; bile ductular hyperplasia; focal lymphocytic interstitial nephritis, meningitis, orchitis; eosinophilic intravascular leucostasis; splenic fibrovascular tag; multifocal eosinophilic capsular orchitis. | Cervical, lung marginal, mesenteric lymph node lymphoid hyperplasia; acute pulmonary emphysema; lung marginal lymph node sinusoidal eosinophilia, histiocytosis; acute tissue congestion; multifocal gastric mucosal lymphoid hyperplasia; multifocal lymphoplasmacytic, eosinophilic capsular mesenteric lymphadenitis; mesenteric lymph node lymphoid sinus eosinophilia; bone marrow eosinophilic myelopoiesis. | Splenic lymphoid hyperplasia. | Balanitis and orchitis could affect reproductive success if they persist to maturity. | |
| 19 (N4645) | Ta | M | C | S | Cervical lymph node, bronchiole-associated lymphoid hyperplasia; multifocal lymphocytic tracheitis, pleuritis; focal pulmonary fibrosis, necrosis; lung marginal lymph node focal lymphoplasmacytic, eosinophilic capsular lymphadenitis, sinusoidal eosinophilia; focal lymphocytic gastritis, lymphocytic capsular adrenal adenitis; splenic fibrovascular tag. | Acute pulmonary oedema, emphysema; multifocal eosinophilic, lymphoplasmacytic interstitial pneumonia; lung marginal, mesenteric lymph node lymphoid hyperplasia; acute tissue congestion; bile ductular hyperplasia; subintimal mineralisation, haematoidin (umbilical artery). | Multifocal bronchiole mineralisation; splenic lymphoid hyperplasia. | Healthy. | |
| 20 (N3583) | Ta | F | J | N | Acute subcutaneous congestion; acute tissue congestion; multifocal lymphocytic perivascular glossitis, endometritis, oophoritis; multifocal eosinophilic capsular mesenteric lymphadenitis, adjacent steatitis; lymphocytic periportal hepatitis with multifocal periportal fibrosis; renal corticomedullary lymphocytic hyperplasia; multifocal lymphocytic perirenal steatitis. | Eosinophilic intravascular leucostasis; multifocal acute eosinophilic steatitis (around cervical lymph node); acute pulmonary congestion, oedema, emphysema; multifocal bronchiole-associated, splenic, uterine, mesenteric lymph node lymphoid hyperplasia; multifocal bronchiolar mineralization; multifocal eosinophilic enteritis. | Multifocal lymphoplasmacytic, eosinophilic interstitial pneumonia, pleuritis; bone marrow eosinophilic myelopoiesis. | Healthy. | |
| 21 (N4355) | Ta | F | J | N | Intravascular eosinophilic leucostasis; diffuse eosinophilic mastitis, gastritis; acute alveolar emphysema; multifocal lymphoplasmacytic, eosinophilic interstitial pneumonia with multifocal lymphoid hyperplasia; multifocal bronchiolar, optic nerve perineurium, renal tubular luminal, cerebral grey matter mineralisation; multifocal lymphocytic ganglionitis in epicardial fat, oesophagitis, gastritis, mesenteric lymphadenitis, steatitis (adjacent to pancreas), transmural cystitis; focal diaphragmatic lymphoplasmacytic myositis, hepatitis, interstitial nephritis; multifocal lymphoplasmacytic, neutrophilic intracapsular hepatitis; diffuse bile stasis (caniculi); multifocal lymphoplasmacytic, eosinophilic cholangitis; splenic haemosiderosis; uterine lymphangiectasia; multifocal lymphoplasmacytic perivascular meningoencephalitis. | Focal lymphoplasmacytic, eosinophilic ulcerative dermatitis; multifocal lymphocytic necrotising panniculitis; acute tissue congestion; multifocal lymphoplasmacytic myocarditis; multifocal myocardial fibrosis; diffuse eosinophilic, lymphoplasmacytic transmural enteritis; diffuse neutrophilic hyperplastic cholangitis; focal eosinophilic cholangitis; pancreatic ductular ectasia; bone marrow eosinophilic myelopoiesis. | Acute pulmonary oedema; focal lymphoplasmacytic, eosinophilic verminous gastritis with pronounced fibrosis (with trematode eggs, perivascular lymphoid hyperplasia); focal neutrophilic, eosinophilic enteritis; splenic lymphoid hyperplasia. | Not gravid; myocardial fibrosis and myocarditis as well as meningoencephalitis could affect diving and feeding success. | |
| 22 (N4529) | Ta | F | J | N | Multifocal lymphoplasmacytic myositis, diaphragmatic myositis; focal histiocytic, eosinophilic cervical lymph node capsular lymphadenitis; diffuse eosinophilic interstitial pneumonia; multifocal bronchiolar, uterine mucosal, renal tubular luminal mineralisation; pulmonary alveolar corpora amylacea; pneumoconiosis (lung, lung marginal lymph node); multifocal lymphocytic myocarditis, transmural cystitis; multifocal lymphoplasmacytic, eosinophilic gastritis; splenic lymphoid hyperplasia; bone marrow eosinophilic myelopoiesis; *Shewonella algae* isolated from the lung. | Multiple white firm foci of lymphoplasmacytic, histiocytic, eosinophilic necrotizing verminous myositis (adjacent to mammary gland, *Crassicauda* sp.); corpora amylacea in mammary acini; cervical lymph node sinusoidal eosinophilic leucostasis; acute pulmonary congestion, emphysema; multifocal lymphocytic interstitial pneumonia with moderate multifocal lymphoid hyperplasia; lung marginal lymph node sinusoidal eosinophilic leucostasis, lymphoid hyperplasia, multifocal eosinophilic, lymphoplasmacytic, histiocytic capsular lymphadenitis; acute tissue congestion; diffuse eosinophilic enteritis. | Intravascular eosinophilic leucostasis; focal gastric mucosal mineralization. | Healthy. | |
| 23 (N4537) | Ta | F | J | N | Eosinophilic intravascular leucostasis; multifocal lymphoplasmacytic, eosinophilic tracheitis, pleuritis with moderate pleural fibrosis (adhesions), lung marginal lymph node capsular lymphadenitis, gastritis, mesenteric lymph node capsular lymphadenitis, transmural cystitis; acute pulmonary congestion, emphysema, oedema; multifocal bronchiolar mineralisation; multifocal bronchiole-associated lymphoid hyperplasia; lung marginal lymph node haemosiderosis, eosinophilic leucostasis; focal lymphocytic myocarditis, periportal hepatitis; multifocal, perivascular lymphoplasmacytic glossitis with mild acute focal muscle necrosis; multifocal lymphoplasmacytic interstitial nephritis. | Focal lymphoplasmacytic myositis; diffuse lymphoplasmacytic, eosinophilic transmural enteritis, peritonitis (with fibrosis), perirenal steatitis; acute tissue congestion; multifocal gastric mucosal lymphoid hyperplasia; multifocal chronic lymphoplasmacytic, eosinophilic gastritis ( with trematode adults, eggs (Brachycladiinae)); adenohypophyseal cysts. | Multifocal lymphoplasmacytic, eosinophilic, neutrophilic bronchointerstitial pneumonia; eosinophilic intravascular leucostasis (bladder); splenic fibrovascular tags with mild multifocal eosinophilic capsular splenitis; bone marrow eosinophilic myelopoiesis. | Pleural adhesions and and fdeding success if they persist tarathyroidism were present, glossitis could affect diving and feeding success. | |
| 24 (N4352) | Ta | M | J | N | Multifocal lymphoplasmacytic, histiocytic cervical lymph node capsular lymphadenitis; focal lymphocytic tracheitis; acute pulmonary oedema, congestion; multifocal bronchiolar mineralisation; multifocal lymphocytic, eosinophilic myocarditis, transmural enteritis, mesenteric lymph node capsular, parenchymal adenitis, capsular hepatitis, subserosal cystitis; multifocal myocardial fibrosis; multifocal renal corticomedullary lymphoid hyperplasia; lymphocytic cortical interstitial nephritis, pyelonephritis; multifocal lymphoplasmacytic capsular splenitis; splenic, cervical lymph node lymphoid hyperplasia. | Acute pulmonary emphysema; multifocal chronic lymphoplasmacytic, predominantly eosinophilic bronchointerstitial pneumonia, pleuritis; acute tissue congestion; eosinophilic intravascular leucostasis; focal lymphocytic, histiocytic, necrotizing enteritis; focal eosinophilic mesenteric lymphadenitis; lymphoplasmacytic, eosinophilic periportal hepatitis with fibrosis, bile ductular hyperplasia; multiple white testicular foci of lymphocytic, eosinophilic, neutrophilic necrotising capsular orchitis with mild fibrosis; bone marrow eosinophilic myelopoiesis. |  | Myocarditis and fibrosis could affect diving and feeding success. | |
| 25 (N4533) | Ta | M | J | N | Multifocal lymphoplasmacytic salivary adenitis, interstitial pneumonia, myocarditis, gastritis, capsular hepatitis, encephalitis, capsular splenitis (variably associated with splenic fibrovascular tags); focal pulmonary fibrosis, mineralisation; acute pulmonary congestion, oedema, emphysema; pulmonary corpora amylacea; focal neutrophilic, eosinophilic, histiocytic necrotising lung marginal lymph node lymphadenitis with lymphoid hyperplasia, parenchymal, capsular mesenteric lymphadenitis (with nematode larvae); acute tissue congestion; multifocal gastric mucosal lymphoid hyperplasia; multifocal gastric mucosal, renal tubular luminal mineralisation; multifocal lymphoplasmacytic necrotising glossitis; multifocal eosinophilic enteritis; multifocal histiocytic, eosinophilic, lymphoplasmacytic periportal hepatitis. | Mesenteric lymph node, splenic lymphoid hyperplasia; acute tissue congestion; multifocal chronic lymphoplasmacytic gastritis (with adult trematode, eggs); multifocal lymphocytic, eosinophilic, histiocytic necrotising mural enteritis. | Multifocal bronchiole-associated lymphoid tissue lymphoid hyperplasia. | Glossitis could affect feeding success. | |
| 26 (N3581) | Ta | F | J | S | Cutaneous laceration; multifocal lymphoplasmacytic, eosinophilic cervical lymph node capsular lymphadenitis, perinodal steatitis, enteritis, endometritis; multifocal lymphocytic tracheitis, perivascular gastritis; multifocal bronchiolar mineralisation; focal myocardial fibrosis; multifocal peritoneal haemorrhage; mild serous ascites; acute tissue congestion; splenic fibrovascular tag with focal eosinophilic capsular splenitis; Anisakidae in the intestinal lumen. | Cervical, mesenteric lymph node lymphoid hyperplasia; multifocal lymphoplasmacytic, eosinophilic necrotising perithyroid steatitis, interstitial pneumonia, diaphragmatic serositis, gastritis, capsular mesenteric lymphadenitis, periportal hepatitis (with trematode eggs); firm white focal subpleural fibrosis, necrosis; acute alveolar, subpleural emphysema; multifocal alveolar histiocytosis; moderate acute tissue congestion; moderate multifocal eosinophilic enteritis; intravascular eosinophilic leucostasis (mesenteric lymph node); periportal, subcapsular fibrosis with bile ductular hyperplasia. | Acute pulmonary oedema; eosinophilic leucostasis; multifocal lymphocytic, eosinophilic capsular splenitis. | Myocardial fibrosis could affect diving and feeding success. | |
| 27 (N4534) | Ta | F | J | S | Diffuse lymphocytic, eosinophilic, histiocytic tracheitis; multifocal bronchiole-associated, gastric mucosal lymphoid hyperplasia; eosinophilic intravascular leucostasis; multifocal lymphocytic, eosinophilic myocarditis, enteritis; acute focal subendocardial haemorrhage; multifocal lymphocytic gastritis, capsular adrenal adenitis, interstitial cortical nephritis, meningitis; focal lymphoplasmacytic necrotising enteritis with fibrosis; splenic fibrovascular tags; bone marrow eosinophilic myelopoiesis. | Focal acute skeletal muscle haemorrhage; acute tissue congestion; multifocal eosinophilic, histiocytic necrotising bronchopneumonia; multifocal lymphoplasmacytic, eosinophilic interstitial pneumonia, marginal lymph node capsular lymphadenitis; acute diffuse pulmonary oedema, emphysema; multifocal bronchiolar mineralisation; lung marginal lymph node haemosiderosis, lymphoid atrophy; multifocal chronic lymphocytic, eosinophilic necrotizing gastritis (with mucosal mineralisation, trematode eggs); multifocal gastric mucosal lymphoid hyperplasia; mesenteric lymph node haemosiderosis; lymphoplasmacytic periportal, multifocal hepatitis. |  | Healthy. | |
| 28 (N4535) | Ta | F | J | S | Diffuse lymphoplasmacytic, eosinophilic, histiocytic interstitial pneumonia; acute pulmonary oedema; multifocal bronchiolar mineralisation; lung marginal lymph node lymphoid depletion; eosinophilic intravascular leucostasis; focal lymphoplasmacytic endocarditis; multifocal lymphoplasmacytic, eosinophilic gastritis, arteritis; focal granulomatous enteritis; diffuse eosinophilic enteritis; multifocal renal corticomedullary lymphocytic hyperplasia; splenic fibrovascular tags. | Multifocal pulmonary emphysema; lung marginal lymph node eosinophilic sinusoidal leucostasis; acute tissue congestion; bone marrow eosinophilic myelopoiesis. | Multifocal chronic lymphoplasmacytic, eosinophilic necrotizing gastritis (with an adult trematode, eggs), mucosal mineralisation of the overlying mucosa, lymphoid hyperplasia; adenohypophyseal cysts | Healthy. | |
| 29 (N4541) | Ta | M | J | S | Eosinophilic intravascular leucostasis; multifocal lymphoplasmacytic salivary adenitis; cervical lymph node lymphoid hyperplasia; multifocal lymphocytic, eosinophilic interstitial pneumonia, pleuritis, lung marginal lymph node capsular lymphadenitis, capsular splenitis; multifocal bronchiolar mineralisation; multifocal lymphocytic mural gastritis; multifocal eosinophilic enteritis; pancreatic ductular ectasia; splenic fibrovascular tags; multifocal lymphoplasmacytic, histiocytic perivascular cerebral encephalitis; cerebral neuronal satellitosis. | Acute pulmonary emphysema; lung marginal lymph node pneumoconiosis; multifocal lymphoplasmacytic myocarditis; focal myocardial fibrosis; acute tissue congestion; diffuse eosinophilic, lymphoplasmacytic necrotizing parenchymal, capsular mesenteric lymphadenitis (with a nematode larva); subcapsular hepatic bile ductular hyperplasia; focal lymphoplasmacytic necrotizing subserosal cystitis with laminated non-birefringent crystals. |  | Myocarditis and myocardial fibrosis could affect diving and feeding success. | |
| 30 (N3575) | Ta | F | A | N | Multifocal lymphocytic, neutrophilic salivary lymphadenitis, mesenteric lymphadenitis (with haematoidin-laden macrophages and crystalline material); multifocal lymphoplasmacytic, eosinophilic skeletal muscle arteritis, subserosal gastritis, corticomedullary interstitial nephritis; multifocal lymphoplasmacytic perivascular myositis, epicarditis, diaphragmatic serositis, glossitis, capsular mesenteric lymphadenitis; acute pulmonary emphysema; eosinophilic diffuse interstitial pneumonia; multiple tan subpleural foci of lymphoplasmacytic interstitial pneumonia; multifocal bronchiolar mineralisation; multifocal myocardial fibrosis; mild dental attrition; a few missing maxillary teeth; healed tongue tip amputation; focal lymphoplasmacytic periportal hepatitis (with a trematode egg); splenic fibrovascular tags; partially mineralised ovarian corpus luteum. | Extensive facial cutaneous haemorrhage; cervical, mesenteric lymph node lymphoid hyperplasia; acute laryngeal haemorrhage; acute pulmonary oedema; acute tissue congestion; eosinophilic intravascular leucostasis; multifocal gastric mucosal mineralisation; multifocal lymphoplasmacytic, eosinophilic transmural enteritis, endometritis, metritis; multifocal eosinophilic mesenteric lymphadenitis; incomplete left uterine horn involution. | Multifocal chronic lymphoplasmacytic necrotizing gastritis (with trematode eggs); periportal fibrosis with bile ductular hyperplasia, haemosiderosis. | Possible abortion; myocardial fibrosis could affect diving and feeding success. | |
| 31 (N3578) | Ta | F | A | N | Cutaneous linear lacerations; focal superficial skin ulcer (24x15mm) on ventral midline; multifocal lymphocytic, histiocytic, neutrophilic salivary adenitis; cervical lymph node lymphoid hyperplasia; multifocal bronchiolar mineralization; multifocal myocardial fibrosis; focal lymphoplasmacytic, eosinophilic perivascular glossitis, capsular adrenal adenitis; multifocal lymphocytic gastritis, perivascular cystitis; multifocal eosinophilic ulcerative gastritis; subcapsular bile ductular hyperplasia with fibrosis; renal corticomedullary lymphoid hyperplasia; mineralised focus in ovarian stroma. | Acute tissue congestion; eosinophilic intravascular leucostasis; focal lymphoplasmacytic, eosinophilic interstitial pneumonia (with interstitial fibrosis), diaphragmatic myositis (with fibrosis), capsular mesenteric lymphadenitis (with lymphoid hyperplasia, lymphangiectasia, sinusoidal eosinophilia), periportal hepatitis; multifocal eosinophilic enteritis; multifocal lymphocytic interstitial nephritis; fibrosis around the renal arcuate arteries. | Acute pulmonary emphysema, oedema; diffuse lymphoplasmacytic, histiocytic, eosinophilic transmural multifocal metritis; bone marrow eosinophilic myelopoiesis. | Myocardial fibrosis could affect diving and feeding success; metritis could affect reproductive success. | |
| 32 (N4339) | Ta | F | A | N | Multifocal subcutaneous, gastric mucosal haemorrhage; one malpositioned tooth; multifocal gastric mucosal, pancreatic, splenic, mammary gland lymphoid hyperplasia; multifocal lymphoplasmacytic perivascular myositis (blowhole), glossitis; multifocal subpleural, myocardial fibrosis; multifocal lymphoplasmacytic, eosinophilic tonsillitis, gastritis, interstitial pneumonia; acute pulmonary oedema; multifocal bronchiolar mineralisation; serosanguinous hydropericardium; acute tissue congestion; multifocal transmural eosinophilic enteritis; bile ductular hyperplasia; multifocal lymphoplasmacytic renal cortical arteritis; renal haemosiderosis; splenic fibrovascular tag. | Dental attrition; multifocal lymphoplasmacytic capsular nephritis; lymphoplasmacytic, eosinophilic periportal hepatitis; eosinophilic intravascular leucostasis; multifocal eosinophilic necrotizing endometritis (trematode egg in the lumen); multifocal neutrophilic necrotizing splenitis; bone marrow eosinophilic myelopoiesis. | Multifocal neutrophilic, eosinophilic bronchopneumonia. | Lactating, not gravid; endometritis could affect reproductive success; myocardial fibrosis could affect diving and feeding success. | |
| 33 (N3574) | Ta | M | A | N | Multifocal lymphoplasmacytic, eosinophilic salivary adenitis, cervical lymph node capsular lymphadenitis and splenitis, periportal hepatitis (with bile ductular hyperplasia, moderate fibrosis), gastritis, small intestinal arteritis; focal lymphoplasmacytic perivascular myositis; cervical lymph node lymphoid hyperplasia, sinusoidal eosinophilia; multifocal bronchiolar mineralisation; acute pulmonary oedema, emphysema; malposition of maxillary teeth; eosinophilic intravascular leucostasis; multifocal eosinophilic enteritis (with multifocal mucosal, submucosal, subserosal mineralisation); renal corticomedullary, splenic lymphoid hyperplasia; white foci of lymphoplasmacytic, histiocytic capsular orchitis, hepatitis; focal perivascular lymphocytic scleritis; Anisakidae in the intestinal lumen. | Multifocal pulmonary interstitial fibrosis; acute tissue congestion; focal myocardial fibrosis; multifocal lymphoplasmacytic subserosal gastritis. | Focal chronic lymphoplasmacytic, eosinophilic parasitic gastritis (with trematode eggs). | Myocardial fibrosis could affect diving and feeding success. | |
| 34 (N3590) | Ta | M | A | N | Two puncture wounds (1cm diameter) left ventral neck; lacerations between flippers (< 8cm long); 9 linear healed scars on left lateral side (13cm long); single large subcutaneous fibrous nodule (6x4x4cm) cranial to fluke; lymphoplasmacytic dermatitis, ulcerative oesophagitis, perivascular gastritis, optic nerve perineuritis, interstitial thyroiditis; multifocal lymphoplasmacytic, rarely eosinophilic perivascular pharyngitis; multifocal lymphocytic, eosinophilic cervical, bronchial, mesenteric lymph node capsular lymphadenitis (with eosinophilic sinusoidal leucostasis), bronchitis (with interstitial fibrosis); acute pulmonary congestion oedema, emphysema; multifocal acute pulmonary haemorrhage; multifocal bronchiolar mineralisation; multifocal pulmonary pneumoconiosis; multifocal perivascular myocardial fibrosis; acute focal ulcerative glossitis, pharyngitis; acute pharyngeal petechiae; intravascular eosinophilic leucostasis; focal granulomatous enteritis; periportal fibrosis; renal corticomedullary lymphoid hyperplasia; mineralized foci in renal tubular lumina, optic nerve; focal eosinophilic, necrotising granulomatous bronchial lymphadenitis. | Cervical, lung marginal lymph node lymphoid hyperplasia; multifocal alveolar histiocytosis; lung marginal lymph node haemosiderosis; acute tissue congestion; bronchial, mesenteric lymph node pneumoconiosis; periportal haemosiderosis; splenic fibrovascular tags; two accessory spleens; multifocal splenic lymphocytic capsular lymphadenitis. | Multifocal eosinophilic, neutrophilic, lymphoplasmacytic bronchointerstitial pneumonia; marked dental attrition; healed right cranial mandibular fracture; mesenteric lymphangiectasia; multifocal eosinophilic granulomatous mesenteric lymphadenitis; acute multifocal splenic capsular, parenchymal haemorrhage; focal histiocytic, neutrophilic, lymphoplasmacytic, eosinophilic haemorrhagic balanitis with large numbers of haemosiderin-laden macrophages. | Myocardial fibrosis could affect diving and feeding success; balanitis could affect breeding success. | |
| 35 (N4643) | Ta | F | A | S | Multifocal mammary, bronchiolar, ovarian stroma, optic nerve mineralisation; mammary corpora amylacea; multifocal plasmacytic sialoadenitis; multifocal lymphoplasmacytic cervical lymph node capsular lymphadenitis (with lymphoid hyperplasia, haemosiderosis), oesophagitis, gastritis, interstitial nephritis, oophoritis, transmural metritis, optic perineuritis; intravascular eosinophilic leucostasis); multifocal eosinophilic, lymphocytic interstitial pneumonia, lung marginal, mesenteric lymph node capsular lymphadenitis, transmural enteritis, capsular splenitis; pulmonary, lung marginal lymph node pneumoconiosis; focal gastric mucosal lymphoid hyperplasia; multifocal to diffuse eosinophilic mesenteric lymphadenitis; splenic lymphoid hyperplasia; splenic fibrovascular tag; bone marrow eosinophilic myelopoiesis. | Diffuse pulmonary interstitial fibrosis; acute pulmonary congestion, oedema; acute tissue congestion; multifocal lymphoplasmacytic, eosinophilic transmural gastritis; periportal, subcapsular bile ductular hyperplasia with multifocal periportal fibrosis; hepatic lipofuschinosis. | Diffuse squamous hyperplasia of the palatine mucosa with multifocal mineralisation of underlying submucosal connective tissue. | Oophoritis and metritis could affect reproductive success; oral lesion could affect feeding success. | |
| 36 (N4530) | Sb | F | C | N | Multifocal bronchiolar, hepatic , renal tubular luminal mineralisation; intravascular eosinophilic leucostasis; multifocal lymphocytic eosinophilic interstitial pneumonia; focal gastric submucosal lymphoid hyperplasia, focal lymphoplasmacytic gastritis; mesenteric lymph node, subcapsular hepatic lymphangiectasia; haemosiderosis, lymphoid hyperplasia; subcapsular bile ductular hyperplasia; diffuse eosinophilic to lymphoplasmacytic capsular adrenal adenitis. | Cervical, lung marginal, sublumbar lymph node lymph node paracortical lymphoid hyperplasia; acute diffuse haemorrhage (tracheal submucosa); acute pulmonary congestion, oedema; multifocal neutrophilic, eosinophilic interstitial pneumonia with focal lymphoid hyperplasia; pulmonary alveolar histiocytosis; acute tissue congestion; multifocal eosinophilic, lymphoplasmacytic cholangitis (with severe epithelial hyperplasia), capsular nephritis; focal eosinophilic arteritis (renal arcuate arteries) with moderate concentric fibrosis; splenic haemosiderosis; focal subserosal lymphocytic metritis. | Large accessory spleen; splenic lymphoid hyperplasia. | Healthy. | |
| 37 (N4531) | Sp | F | J | N | Multifocal eosinophilic verminous bronchiolitis; multifocal bronchiolar mineralisation; acute lung marginal lymph node congestion; multifocal lymphoplasmacytic, eosinophilic lung marginal, mesenteric lymph node capsular lymphadenitis, perivascular subserosal enteritis, perivascular oophoritis, metritis, uterine arteritis; diffuse lymphocytic endometritis. | Acute pulmonary oedema, emphysema; multiple firm white foci of histiocytic, lymphoplasmacytic, eosinophilic necrotizing bronchointerstitial pneumonia, pleuritis with lymphoid hyperplasia; intravascular eosinophilic leucostasis; multifocal lymphocytic interstitial cystitis, necrotising myositis. | Lung marginal lymph node medullary sinus histiocytosis; hepatic lipofuschinosis; splenic lymphoid hyperplasia. | Reproductive tract lesions could affect reproductive success if they persisted to maturity; myositis could affect locomotion, feeding success. | |
| 38 (N4542) | Sp | M | J | N | Multifocal lymphoplasmacytic myositis; cervical lymph node lymphoid hyperplasia, medullary plasmacytosis; multifocal eosinophilic, lymphoplasmacytic interstitial pneumonia (with interstitial fibrosis, pleuritis, pleural fibrovascular tags), lung marginal lymph node capsular lymphadenitis (with sinus eosinophilia, histiocytosis, lymphoid hyperplasia), mesenteric capsular lymphadenitis, capsular orchitis; multifocal bronchiolar, renal interstitial, gastric mucosal mineralisation; multifocal pulmonary pneumoconiosis; multifocal perivascular myocardial fibrosis; acute tissue congestion; focal lymphoplasmacytic mural gastritis, transmural cystitis; multifocal eosinophilic enteritis; multifocal lymphocytic, histiocytic, eosinophilic cholangitis with moderate bile ductular hyperplasia, large bile duct epithelial hyperplasia, moderate periportal fibrosis; focal cerebral acute haemorrhage; *Photobacterium damselae*, *Shewanella putrefaciens* isolated from the lung. | Acute pulmonary oedema; alveolar histiocytosis; segmental coronary arteriolosclerosis; multifocal lymphocytic hepatic arteritis; focal plasmacytic, eosinophilic funiculitis. | Intravascular eosinophilic leucostasis. | Myocardial fibrosis could affect diving and feeding success; orchitis and funiculitis could affect reproductive success if it persists to maturity. | |
| 39 (N4341) | Sp | M | A | N | Multifocal bronchiolar mineralisation; hepatic eosinophilic intravascular leucostasis; acute hepatic congestion; bile ductular ectasia; focal renal cortical fibrosis; splenic lymphoid hyperplasia; multiple white testicular foci of acute eosinophilic capsular orchitis. | Small white firm foci of pulmonary fibrosis; pulmonary emphysema; multifocal eosinophilic bronchointerstitial pneumonia; multiple white foci of lymphocytic, eosinophilic capsular nephritis. | Multifocal chronic peritonitis; large accessory spleen. | Healthy. | |
| 40 (N3584) | Sp | M | A | N | Cutaneous net marks; puncture wound behind blowhole (12-14mm diameter); multifocal verminous pulmonary fibrosis, necrosis; multifocal bronchiolar mineralisation; acute tissue congestion; multifocal bronchiole-associated lymphoid hyperplasia; multifocal lymphoplasmacytic, histiocytic interstitial pneumonia; alveolar histiocytosis; several fractured and malpositioned cranial mandibular teeth; focal lymphocytic capsular mesenteric lymphadenitis; two accessory spleens; splenic fibrovascular tags; focal lymphocytic, neutrophilic capsular epididymitis. | Two (10-11mm) cutaneous tan foci below dorsal fin - granulomatous, ulcerative dermatitis with pigmentary incontinence, dermal, subcutaneous fibrosis with large numbers of intralesional fungi and Gram positive cocci on skin surface lLobomycosis); cervical lymph node lymphoid hyperplasia; acute pulmonary oedema, emphysema; moderate dental attrition; focal lymphoplasmacytic; multiple peritoneal adhesions. | Multifocal eosinophilic necrotising bronchointerstitial pneumonia with mild bronchiectasis, haemosiderosis; small fibrovascular pleural tags. | Healthy. | |

^+^ ID refers to Case and (Port Elizabeth Museum accession) numbers

^#^ Species refers to *Tursiops aduncus* (Ta) or *Sousa plumbea* (Sp)

^Sex refers to male (M) or female (F)

*Age refers to a calf <2 years old (C), juvenile 2-12 years old (J) or adult >12 years old (A)

^##^Region refers to dolphins collected from the northern (N) or southern (S) region
