# Supplementary material for: A Systematic Health Assessment of Indian Ocean Bottlenose (Tursiops aduncus) and Indo-Pacific Humpback (Sousa plumbea) Dolphins Incidentally Caught in Shark Nets off the KwaZulu-Natal Coast, South Africa
Source: PLoS One. 2014 Sep 9;9(9):e107038. doi: 10.1371/journal.pone.0107038 (PMC4159300; doi:10.1371/journal.pone.0107038)
Supplement: Table S2 — Complete pathological findings for indicating occurrence (lesion/number of organ evaluated) and percentage per species, age group, and region (for both species combined). (DOCX) [file pone.0107038.s002.docx]

**Table S2: Complete pathological findings for indicating occurrence (lesion/number of organ evaluated) and percentage per species, age group, and region (for both species combined).**

| **Lesion** | **Total** | | | | | ***T. aduncus*** | | | | | | | | | ***S. chinensis*** | | | | | **Region** | |
| --- | --- | --- | --- | --- | --- | --- | --- | --- | --- | --- | --- | --- | --- | --- | --- | --- | --- | --- | --- | --- | --- |
|  |  | | | | | Calf | | | Juvenile | | | Adult | | | Calf | | Juvenile | | Adult | North | South |
| **Pneumonia (total)** | 37/40 | | | | | 17/19 | | | 10/10 | | | 5/6 | | | 1/1 | | 2/2 | | 2/2 | 27/30 | 10/10 |
|  | 93% | | | | | 89% | | | 100% | | | 83% | | | 100% | | 100% | | 100% | 90% | 100% |
| **Mild to severe, multifocal eosinophilic and lymphoplasmacytic parasitic pneumonia** | 8/40 | | | | | 6/19 | | | 0/10 | | | 1/6 | | | 0/1 | | 0/2 | | 1/2 | 6/30 | 2/10 |
|  | 20% | | | | | 32% | | | 0% | | | 17% | | | 0% | | 0% | | 50% | 20% | 20% |
| **Mild to moderate, multifocal to diffuse eosinophilic and variably lymphoplasmacytic pneumonia** | 34/40 | | | | | 15/19 | | | 9/10 | | | 5/6 | | | 1/1 | | 2/2 | | 2/2 | 24/30 | 10/10 |
|  | 85% | | | | | 79% | | | 90% | | | 83% | | | 100% | | 100% | | 100% | 80% | 100% |
| **Mild to moderate, multifocal lymphoplasmacytic pneumonia** | 3/40 | | | | | 2/19 | | | 1/10 | | | 0/6 | | | 0/1 | | 0/2 | | 0/2 | 3/30 | 0/10 |
|  | 8% | | | | | 11% | | | 10% | | | 0% | | | 0% | | 0% | | 0% | 10% | 0% |
| **Mild to moderate, multifocal eosinophilic and lymphoplasmacytic tracheo-bronchitis** | 12/40 | | | | | 8/19 | | | 4/10 | | | 0/6 | | | 0/1 | | 0/2 | | 0/2 | 7/30 | 5/10 |
|  | 30% | | | | | 42% | | | 40% | | | 0% | | | 0% | | 0% | | 0% | 23% | 50% |
| **Moderate to severe, multifocal to diffuse acute alveolar oedema** | 32/40 | | | | | 16/19 | | | 8/10 | | | 6/6 | | | 1/1 | | 1/2 | | 0/2 | 24/30 | 8/10 |
|  | 80% | | | | | 84% | | | 80% | | | 100% | | | 100% | | 100% | | 0% | 80% | 80% |
| **Moderate to severe, multifocal to diffuse alveolar emphysema** | 32/40 | | | | | 17/19 | | | 9/10 | | | 4/6 | | | 0/1 | | 1/2 | | 1/2 | 24/30 | 8/10 |
|  | 80% | | | | | 89% | | | 90% | | | 67% | | | 0% | | 50% | | 50% | 80% | 80% |
| **Mild to severe, multifocal follicular lymphoid hyperplasia** | 18/40 | | | | | 9/19 | | | 6/10 | | | 0/6 | | | 1/1 | | 1/2 | | 1/2 | 14/30 | 4/10 |
|  | 45% | | | | | 47% | | | 60% | | | 0% | | | 100% | | 50% | | 50% | 47% | 40% |
| **Mild to moderate, multifocal, bronchiolar mucosal mineralization** | 33/40 | | | | | 16/19 | | | 8/10 | | | 5/6 | | | 1/1 | | 2/2 | | 1/2 | 25/30 | 8/10 |
|  | 83% | | | | | 84% | | | 80% | | | 83% | | | 100% | | 100% | | 50% | 83% | 80% |
| **Mild, multifocal pneumoconiosis** | 3/40 | | | | | 0/19 | | | 0/10 | | | 2/6 | | | 0/1 | | 1/2 | | 0/2 | 2/30 | 1/10 |
|  | 8% | | | | | 0% | | | 0% | | | 33% | | | 0% | | 50% | | 0% | 7% | 10% |
| **Mild to moderate, multifocal alveolar macrophage hyperplasia (histiocytosis)** | 6/40 | | | | | 3/19 | | | 0/10 | | | 1/6 | | | 1/1 | | 1/2 | | 0/2 | 6/30 | 0/10 |
|  | 15% | | | | | 16% | | | 0% | | | 17% | | | 100% | | 50% | | 0% | 20% | 0% |
| **Small numbers of alveolar *Corpora amylacea*** | 2/40 | | | | | 0/19 | | | 2/10 | | | 0/6 | | | 0/1 | | 0/2 | | 0/2 | 2/30 | 0/10 |
|  | 5% | | | | | 0% | | | 20% | | | 0% | | | 0% | | 0% | | 0% | 7% | 0% |
| **Mild to moderate, multifocal to diffuse, eosinophilic to lymphoplasmacytic pleuritis** | 12/40 | | | | | 6/19 | | | 4/10 | | | 0/6 | | | 0/1 | | 2/2 | | 0/2 | 9/30 | 3/10 |
|  | 30% | | | | | 32% | | | 40% | | | 0% | | | 0% | | 100% | | 0% | 30% | 30% |
| **Mild multifocal pleural fibrosis** | 4/40 | | | | | 1/19 | | | 1/10 | | | 0/6 | | | 0/1 | | 1/2 | | 1/2 | 4/30 | 0/10 |
|  | 10% | | | | | 5% | | | 10% | | | 0% | | | 0% | | 50% | | 50% | 13% | 0% |
| **Pulmonary haemosiderosis** | 1/40 | | | | | 0/19 | | | 0/10 | | | 0/6 | | | 0/1 | | 0/2 | | 1/2 | 1/30 | 0/10 |
|  | 3% | | | | | 0% | | | 0% | | | 0% | | | 0% | | 0% | | 50% | 3% | 0% |
| **Mild multifocal lymphoplasmacytic and variably eosinophilic glossitis** | | | 10/34 | | 3/19 | | | 3/8 | | | 4/5 | | | 0/1 | | | 0/0 | | 0/1 | 9/25 | 1/9 |
|  | | | 29% | | 16% | | | 38% | | | 80% | | | 0% | | | 0% | | 0% | 36% | 11% |
| **Lingual muscle sarcosystosis** | | | 1/34 | | 1/19 | | | 0/8 | | | 0/5 | | | 0/1 | | | 0/0 | | 0/1 | 0/25 | 1/9 |
|  | | | 3% | | 5% | | | 0% | | | 0% | | | 0% | | | 0% | | 0% | 0% | 11% |
| **Mild lymphoplasmacytic pharyngitis** | | | 5/32 | | 4/14 | | | 0/10 | | | 1/5 | | | 0/1 | | | 0/1 | | 0/1 | 5/24 | 0/8 |
|  | | | 16% | | 29% | | | 0% | | | 20% | | | 0% | | | 0% | | 0% | 21% | 0% |
| **Mild multifocal lymphoplasmacytic and variably eosinophilic sialoadenitis** | | | 8/32 | | 2/14 | | | 2/10 | | | 4/5 | | | 0/1 | | | 0/1 | | 0/1 | 6/24 | 2/8 |
|  | | | 25% | | 14% | | | 20% | | | 80% | | | 0% | | | 0% | | 0% | 25% | 25% |
| **Peyer’s patches present in intestine** | | | 22/40 | | 11/19 | | | 6/10 | | | 3/6 | | | 1/1 | | | 1/2 | | 0/2 | 19/30 | 3/10 |
|  | | | 55% | | 58% | | | 60% | | | 50% | | | 100% | | | 50% | | 0% | 63% | 30% |
| **Mild multifocal variably eosinophilic and lymphoplasmacytic oesophagitis** | | | 4/27 | | 2/12 | | | 0/8 | | | 2/3 | | | 0/1 | | | 0/1 | | 0/2 | 3/19 | 1/8 |
|  | | | 15% | | 17% | | | 0% | | | 67% | | | 0% | | | 0% | | 0% | 16% | 13% |
| **Mild multifocal lymphoplasmacytic and eosinophilic gastritis** | | | 28/38 | | 11/19 | | | 8/9 | | | 6/6 | | | 1/1 | | | 1/1 | | 1/2 | 20/28 | 8/10 |
|  | | | 74% | | 58% | | | 89% | | | 100% | | | 100% | | | 100% | | 50% | 71% | 80% |
| **Moderate to severe multifocal parasitic gastritis (trematodes)** | | | 12/38 | | 5/19 | | | 5/9 | | | 2/6 | | | 0/1 | | | 0/1 | | 0/2 | 8/28 | 4/10 |
|  | | | 32% | | 26% | | | 56% | | | 33% | | | 0% | | | 0% | | 0% | 29% | 40% |
| **Mild to severe multifocal to diffuse variably lymphoplasmacytic and eosinophilic enteritis** | | | 27/40 | | 9/19 | | | 10/10 | | | 6/6 | | | 0/1 | | | 2/2 | | 0/2 | 21/30 | 6/10 |
|  | | | 68% | | 47% | | | 100% | | | 100% | | | 0% | | | 100% | | 0% | 70% | 60% |
| **Tonsillar lymphoid follicles present** | | | 22/32 | | 11/14 | | | 7/10 | | | 3/5 | | | 0/1 | | | 0/1 | | 1/1 | 17/24 | 5/8 |
|  | | | 69% | | 79% | | | 70% | | | 60% | | | 0% | | | 0% | | 100% | 71% | 63% |
| **Mild to moderate lymphoplasmacytic and variably eosinophilic multifocal hepatitis** | | | 5/39 | | 3/19 | | | 2/10 | | | 0/6 | | | 0/1 | | | 0/2 | | 0/1 | 4/29 | 1/10 |
|  | | | 13% | | 16% | | | 20% | | | 0% | | | 0% | | | 0% | | 0% | 14% | 10% |
| **Mild to moderate lymphoplasmacytic and eosinophilic periportal hepatitis** | | | 20/39 | | 11/19 | | | 4/10 | | | 5/6 | | | 0/1 | | | 0/2 | | 0/1 | 16/29 | 4/10 |
|  | | | 51% | | 58% | | | 40% | | | 83% | | | 0% | | | 0% | | 0% | 55% | 40% |
| **Mild chronic lymphoplasmacytic and eosinophilic parasitic periportal hepatitis** | | | 2/39 | | 0/19 | | | 1/10 | | | 1/6 | | | 0/1 | | | 0/2 | | 0/1 | 1/29 | 1/10 |
|  | | | 5% | | 0% | | | 10% | | | 17% | | | 0% | | | 0% | | 0% | 3% | 10% |
| **Moderate multifocal lymphoplasmacytic and eosinophilic cholangitis** | | | 4/39 | | 1/19 | | | 1/10 | | | 0/6 | | | 1/1 | | | 1/2 | | 0/1 | 4/29 | 0/10 |
|  | | | 10% | | 5% | | | 5% | | | 0% | | | 100% | | | 50% | | 0% | 14% | 0% |
| **Mild to severe periportal and subcapsular bile ductular hyperplasia** | | | 17/39 | | 7/19 | | | 3/10 | | | 5/6 | | | 1/1 | | | 1/2 | | 0/1 | 12/29 | 5/10 |
|  | | | 44% | | 37% | | | 30% | | | 83% | | | 100% | | | 50% | | 0% | 41% | 50% |
| **Mild to moderate periportal fibrosis** | | | 10/39 | | 2/19 | | | 3/10 | | | 4/6 | | | 0/1 | | | 1/2 | | 0/1 | 7/29 | 3/10 |
|  | | | 26% | | 11% | | | 30% | | | 67% | | | 0% | | | 50% | | 0% | 24% | 30% |
| **Mild to moderate multifocal hepatic capsular fibrosis** | | | 3/39 | | 0/19 | | | 1/10 | | | 2/6 | | | 0/1 | | | 0/2 | | 0/1 | 2/29 | 1/10 |
|  | | | 8% | | 0% | | | 10% | | | 33% | | | 0% | | | 0% | | 0% | 7% | 10% |
| **Mild multifocal lymphoplasmacytic and eosinophilic pancreatitis** | | | 2/35 | | 2/17 | | | 0/9 | | | 0/5 | | | 0/1 | | | 0/2 | | 0/1 | 2/26 | 0/9 |
|  | | | 6% | | 12% | | | 0% | | | 0% | | | 0% | | | 0% | | 0% | 8% | 0% |
| **Mild multifocal splenic capsular fibrosis** | | 18/39 | | 7/19 | | | 6/10 | | | 4/5 | | | 0/1 | | | 0/2 | | 1/2 | | 10/29 | 8/10 |
|  | | 46% | | 37% | | | 60% | | | 80% | | | 0% | | | 0% | | 50% | | 34% | 80% |
| **Mild to moderate multifocal variably lymphoplasmacytic and eosinophilic splenic serositis** | | 12/39 | | 4/19 | | | 4/10 | | | 4/5 | | | 0/1 | | | 0/2 | | 0/2 | | 8/29 | 4/10 |
|  | | 31% | | 21% | | | 40% | | | 80% | | | 0% | | | 0% | | 0% | | 28% | 40% |
| **Mild to moderate multifocal splenic lymphoid hyperplasia** | | 7/39 | | 2/19 | | | 2/10 | | | 3/5 | | | 0/1 | | | 0/2 | | 0/2 | | 6/29 | 1/10 |
|  | | 18% | | 11% | | | 20% | | | 60% | | | 0% | | | 0% | | 0% | | 21% | 10% |
| **Mild to moderate multifocal eosinophilic and variably lymphoplasmacytic cervical lymph node serositis** | | 10/39 | | 4/18 | | | 3/10 | | | 3/6 | | | 0/1 | | | 0/2 | | 0/2 | | 6/29 | 4/10 |
|  | | 26% | | 22% | | | 30% | | | 50% | | | 0% | | | 0% | | 0% | | 21% | 40% |
| **Mild multifocal cervical lymph node lymphoid hyperplasia** | | 8/39 | | 3/18 | | | 1/10 | | | 2/6 | | | 1/1 | | | 1/2 | | 0/2 | | 4/29 | 4/10 |
|  | | 21% | | 17% | | | 10% | | | 33% | | | 100% | | | 50% | | 0% | | 14% | 40% |
| **Mild cervical lymph node haemosiderosis** | | 1/39 | | 0/18 | | | 0/10 | | | 1/6 | | | 0/1 | | | 0/2 | | 0/2 | | 0/29 | 1/10 |
|  | | 3% | | 0% | | | 0% | | | 17% | | | 0% | | | 0% | | 0% | | 0% | 10% |
| **Mild to severe multifocal eosinophilic and variably lymphoplasmacytic mesenteric lymph node serositis** | | 19/39 | | 5/19 | | | 5/10 | | | 5/5 | | | 0/1 | | | 2/2 | | 2/2 | | 15/29 | 4/10 |
|  | | 49% | | 26% | | | 50% | | | 100% | | | 0% | | | 100% | | 100% | | 52% | 40% |
| **Mild multifocal mesenteric lymph node lymphoid hyperplasia** | | 6/39 | | 3/19 | | | 2/10 | | | 1/5 | | | 0/1 | | | 0/2 | | 0/2 | | 6/29 | 0/10 |
|  | | 15% | | 16% | | | 20% | | | 20% | | | 0% | | | 0% | | 0% | | 31% | 0% |
| **Mild multifocal mesenteric lymph node lymphoid hypoplasia** | | 1/39 | | 0/19 | | | 0/10 | | | 1/5 | | | 0/1 | | | 0/2 | | 0/2 | | 0/29 | 1/10 |
|  | | 3% | | 0% | | | 0% | | | 20% | | | 0% | | | 0% | | 0% | | 0% | 10% |
| **Mild multifocal mesenteric lymph node haemosiderosis** | | 4/39 | | 1/19 | | | 1/10 | | | 1/5 | | | 1/1 | | | 0/2 | | 0/2 | | 2/29 | 2/10 |
|  | | 10% | | 5% | | | 10% | | | 20% | | | 100% | | | 0% | | 0% | | 7% | 20% |
| **Mild multifocal mesenteric lymph node anthracosis** | | 1/39 | | 0/19 | | | 0/10 | | | 0/5 | | | 0/1 | | | 0/2 | | 1/2 | | 1/29 | 0/10 |
|  | | 3% | | 0% | | | 0% | | | 0% | | | 0% | | | 0% | | 50% | | 7% | 0% |
| **Mild to moderate multifocal eosinophilic and lymphoplasmacytic lung marginal lymph node serositis** | | 14/30 | | 5/13 | | | 5/8 | | | 2/6 | | | 0/1 | | | 2/2 | | 0/0 | | 9/22 | 5/8 |
|  | | 47% | | 38% | | | 63% | | | 33% | | | 0% | | | 100% | | 0% | | 41% | 63% |
| **Mild multifocal marginal lymph node of the lung lymphoid hyperplasia** | | 3/30 | | 1/13 | | | 1/8 | | | 0/6 | | | 0/1 | | | 1/2 | | 0/0 | | 3/22 | 0/8 |
|  | | 10% | | 8% | | | 13% | | | 0% | | | 0% | | | 50% | | 0% | | 14% | 0% |
| **Moderate multifocal marginal lymph node of the lung lymphoid hypoplasia** | | 1/30 | | 0/13 | | | 1/8 | | | 0/6 | | | 0/1 | | | 0/2 | | 0/0 | | 0/22 | 1/8 |
|  | | 3% | | 0% | | | 13% | | | 0% | | | 0% | | | 0% | | 0% | | 0% | 13% |
| **Mild multifocal marginal lymph node of the lung haemosiderosis** | | 3/30 | | 0/13 | | | 2/8 | | | 1/6 | | | 0/1 | | | 0/2 | | 0/0 | | 2/22 | 1/8 |
|  | | 10% | | 0% | | | 25% | | | 17% | | | 0% | | | 0% | | 0% | | 9% | 13% |
| **Mild multifocal marginal lymph node of the lung pneumoconiosis** | | 3/30 | | 0/13 | | | 2/8 | | | 1/6 | | | 0/1 | | | 0/2 | | 0/0 | | 1/22 | 2/8 |
|  | | 10% | | 0% | | | 25% | | | 17% | | | 0% | | | 0% | | 0% | | 9% | 13% |
| **Mild multifocal lymphoplasmacytic thymitis and peri-thymitis** | | 2/21 | | 2/15 | | | 0/4 | | | 0/1 | | | 0/1 | | | 0/0 | | 0/0 | | 1/14 | 1/7 |
|  | | 10% | | 13% | | | 0% | | | 0% | | | 0% | | | 0% | | 0% | | 7% | 14% |
| **Small acini of peri-thymic glandular cells** | | 2/21 | | 0/15 | | | 1/4 | | | 0/1 | | | 1/1 | | | 0/0 | | 0/0 | | 1/14 | 1/7 |
|  | | 10% | | 0% | | | 25% | | | 0% | | | 100% | | | 0% | | 0% | | 7% | 14% |
| **Mild multifocal lymphoplasmacytic and variably eosinophilic oophoritis** | | 5/27 | | 1/14 | | | 2/7 | | | 1/4 | | | 0/1 | | | 1/1 | | 0/0 | | 4/19 | 1/8 |
|  | | 19% | | 7% | | | 29% | | | 25% | | | 0% | | | 100% | | 0% | | 21% | 13% |
| **Mild multifocal ovarian mineralization** | | 3/27 | | 0/14 | | | 0/7 | | | 3/4 | | | 0/1 | | | 0/1 | | 0/0 | | 2/19 | 1/8 |
|  | | 11% | | 0% | | | 0% | | | 75% | | | 0% | | | 0% | | 0% | | 11% | 13% |
| **Mild to moderate multifocal eosinophilic and lymphoplasmacytic endometritis** | | 11/27 | | 4/14 | | | 2/7 | | | 4/4 | | | 0/1 | | | 1/1 | | 0/0 | | 8/19 | 3/8 |
|  | | 41% | | 29% | | | 29% | | | 100% | | | 0% | | | 100% | | 0% | | 42% | 38% |
| **Moderate multifocal eosinophilic parasitic endometritis (trematode)** | | 1/27 | | 0/14 | | | 0/7 | | | 1/4 | | | 0/1 | | | 0/1 | | 0/0 | | 1/19 | 0/8 |
|  | | 4% | | 0% | | | 0% | | | 25% | | | 0% | | | 0% | | 0% | | 5% | 0% |
| **Mild to moderate multifocal eosinophilic and lymphoplasmacytic metritis** | | 6/27 | | 2/14 | | | 0/7 | | | 3/4 | | | 0/1 | | | 1/1 | | 0/0 | | 4/19 | 2/8 |
|  | | 22% | | 14% | | | 0% | | | 75% | | | 0% | | | 100% | | 0% | | 21% | 25% |
| **Mild multifocal lymphoplasmacytic mastitis** | | 3/7 | | 2/3 | | | 1/2 | | | 0/2 | | | 0/0 | | | 0/0 | | 0/0 | | 3/6 | 0/1 |
|  | | 43% | | 66% | | | 50% | | | 0% | | | 0% | | | 0% | | 0% | | 50% | 0% |
| **Mild to moderate multifocal mammary ductular ectasia** | | 5/7 | | 2/3 | | | 1/2 | | | 2/2 | | | 0/0 | | | 0/0 | | 0/0 | | 4/6 | 1/1 |
|  | | 71% | | 66% | | | 50% | | | 100% | | | 0% | | | 0% | | 0% | | 67% | 100% |
| **Small to large numbers of mammary *corpora amylacea*** | | 3/7 | | 0/3 | | | 2/2 | | | 1/2 | | | 0/0 | | | 0/0 | | 0/0 | | 2/6 | 1/1 |
|  | | 43% | | 0% | | | 100% | | | 50% | | | 0% | | | 0% | | 0% | | 33% | 100% |
| **Mild multifocal mammary dystrophic calcification** | | 2/7 | | 1/3 | | | 0/2 | | | 1/2 | | | 0/0 | | | 0/0 | | 0/0 | | 1/6 | 1/1 |
|  | | 29% | | 33% | | | 0% | | | 50% | | | 0% | | | 0% | | 0% | | 17% | 100% |
| **Mild to moderate multifocal eosinophilic and variably lymphoplasmacytic testis and epididymis serositis** | | 6/13 | | 1/5 | | | 1/3 | | | 1/2 | | | 0/0 | | | 1/1 | | 2/2 | | 6/11 | 0/2 |
|  | | 46% | | 20% | | | 33% | | | 50% | | | 0% | | | 100% | | 100% | | 55% | 0% |
| **Mild multifocal lymphoplasmacytic balanitis** | | 2/5 | | 2/4 | | | 0/0 | | | 0/0 | | | 0/0 | | | 0/0 | | 0/1 | | 2/4 | 0/1 |
|  | | 40% | | 50% | | | 0% | | | 0% | | | 0% | | | 0% | | 0% | | 50% | 0% |
| **Mild multifocal lymphoplasmacytic myocarditis** | | 15/39 | | 8/19 | | | 7/10 | | | 0/6 | | | 0/1 | | | 0/1 | | 0/1 | | 11/29 | 4/10 |
|  | | 38% | | 42% | | | 70% | | | 0% | | | 0% | | | 0% | | 0% | | 38% | 40% |
| **Mild multifocal lymphoplasmacytic epicarditis** | | 3/39 | | 2/19 | | | 0/10 | | | 1/6 | | | 0/1 | | | 0/1 | | 0/1 | | 3/29 | 0/10 |
|  | | 8% | | 11% | | | 0% | | | 17% | | | 0% | | | 0% | | 0% | | 10% | 0% |
| **Mild multifocal lymphoplasmacytic endocarditis** | | 7/39 | | 6/19 | | | 1/10 | | | 0/6 | | | 0/1 | | | 0/1 | | 0/1 | | 4/29 | 3/10 |
|  | | 18% | | 32% | | | 10% | | | 0% | | | 0% | | | 0% | | 0% | | 14% | 30% |
| **Mild to moderate multifocal to diffuse myocardial fibrosis** | | 10/39 | | 0/19 | | | 4/10 | | | 5/6 | | | 0/1 | | | 1/1 | | 0/1 | | 8/29 | 2/10 |
|  | | 26% | | 0% | | | 40% | | | 83% | | | 0% | | | 100% | | 0% | | 28% | 20% |
| **Mild segmental coronary arteriolosclerosis** | | 1/39 | | 0/19 | | | 0/10 | | | 0/6 | | | 0/1 | | | 1/1 | | 0/1 | | 1/29 | 0/10 |
|  | | 3% | | 0% | | | 0% | | | 0% | | | 0% | | | 100% | | 0% | | 3% | 0% |
| **Moderate focal endocardial calcification** | | 1/39 | | 1/19 | | | 0/10 | | | 0/6 | | | 0/1 | | | 0/1 | | 0/1 | | 1/29 | 0/10 |
|  | | 3% | | 5% | | | 0% | | | 0% | | | 0% | | | 0% | | 0% | | 3% | 0% |
| **Mild multifocal lymphoplasmacytic adrenal adenitis** | | 7/37 | | 4/18 | | | 1/10 | | | 1/5 | | | 1/1 | | | 0/1 | | 0/2 | | 5/28 | 2/9 |
|  | | 19% | | 22% | | | 10% | | | 20% | | | 100% | | | 0% | | 0% | | 18% | 22% |
| **Mild multifocal acute adrenal capsular haemorrhage** | | 1/37 | | 1/18 | | | 0/10 | | | 0/5 | | | 0/1 | | | 0/1 | | 0/2 | | 1/28 | 0/9 |
|  | | 3% | | 6% | | | 0% | | | 0% | | | 0% | | | 0% | | 0% | | 4% | 0% |
| **Mild multifocal lymphoplasmacytic thyroiditis** | | 2/27 | | 1/13 | | | 0/7 | | | 1/4 | | | 0/1 | | | 0/1 | | 0/1 | | 1/19 | 1/8 |
|  | | 7% | | 8% | | | 0% | | | 25% | | | 0% | | | 0% | | 0% | | 5% | 13% |
| **Mild colloidal goitre** | | 1/27 | | 1/13 | | | 0/7 | | | 0/4 | | | 0/1 | | | 0/1 | | 0/1 | | 1/19 | 0/8 |
|  | | 4% | | 8% | | | 0% | | | 0% | | | 0% | | | 0% | | 0% | | 5% | 0% |
| **Mild multifocal lymphoplasmacytic peri-thyroidal steatitis** | | 2/27 | | 1/13 | | | 1/7 | | | 0/4 | | | 0/1 | | | 0/1 | | 0/1 | | 1/19 | 1/8 |
|  | | 7% | | 8% | | | 14% | | | 0% | | | 0% | | | 0% | | 0% | | 5% | 13% |
| **Single neck muscle sarcosysts** | | 1/27 | | 0/13 | | | 0/7 | | | 0/4 | | | 0/1 | | | 0/1 | | 0/1 | | 0/19 | 1/8 |
|  | | 4% | | 0% | | | 0% | | | 0% | | | 0% | | | 0% | | 0% | | 0% | 13% |
| **Mild multifocal lymphoplasmacytic pituitary adenitis** | | 2/6 | | 2/3 | | | 0/2 | | | 0/0 | | | 0/0 | | | 0/1 | | 0/0 | | 2/4 | 0/2 |
|  | | 33% | | 66% | | | 0% | | | 0% | | | 0% | | | 0% | | 0% | | 50% | 0% |
| **Mild multifocal lymphoplasmacytic pituitary perineuritis** | | 1/6 | | 1/3 | | | 0/2 | | | 0/0 | | | 0/0 | | | 0/1 | | 0/0 | | 1/4 | 0/2 |
|  | | 17% | | 33% | | | 0% | | | 0% | | | 0% | | | 0% | | 0% | | 25% | 0% |
| **Small numbers of small pituitary cysts** | | 1/6 | | 0/3 | | | 2/2 | | | 0/0 | | | 0/0 | | | 1/1 | | 0/0 | | 1/4 | 2/2 |
|  | | 17% | | 0% | | | 100% | | | 0% | | | 0% | | | 100% | | 0% | | 25% | 100% |
| **Mild multifocal lymphoplasmacytic meningo-encephalitis** | | | 7/18 | | 3/9 | | | 4/6 | | | 0/1 | | | 0/1 | | | 0/1 | | 0/0 | 5/10 | 2/8 |
|  | | | 39% | | 33% | | | 67% | | | 0% | | | 0% | | | 0% | | 0% | 50% | 25% |
| **Mild multifocal acute meningeal haemorrhage** | | | 2/18 | | 1/9 | | | 0/6 | | | 0/1 | | | 0/1 | | | 1/1 | | 0/0 | 1/10 | 1/8 |
|  | | | 11% | | 11% | | | 0% | | | 0% | | | 0% | | | 100% | | 0% | 10% | 13% |
| **Severe focal acute peri-dural spinal haemorrhage** | | | 1/18 | | 0/9 | | | 1/6 | | | 0/1 | | | 0/1 | | | 0/1 | | 0/0 | 1/10 | 0/8 |
|  | | | 6% | | 0% | | | 17% | | | 0% | | | 0% | | | 0% | | 0% | 0% | 0% |
| **Mild multifocal neuronal satellitosis** | | | 2/18 | | 1/9 | | | 1/6 | | | 0/1 | | | 0/1 | | | 0/1 | | 0/0 | 1/10 | 1/8 |
|  | | | 11% | | 11% | | | 17% | | | 0% | | | 0% | | | 0% | | 0% | 10% | 13% |
| **Moderate locally extensive cerebellar herniation** | | | 1/18 | | 1/9 | | | 0/6 | | | 0/1 | | | 0/1 | | | 0/1 | | 0/0 | 1/10 | 0/8 |
|  | | | 6% | | 11% | | | 0% | | | 0% | | | 0% | | | 0% | | 0% | 10% | 0% |
| **Mild to moderate multifocal lymphoplasmacytic interstitial nephritis** | | | 13/39 | | 5/19 | | | 4/10 | | | 3/6 | | | 1/1 | | | 0/2 | | 0/1 | 11/29 | 2/10 |
|  | | | 33% | | 26% | | | 40% | | | 50% | | | 100% | | | 0% | | 0% | 38% | 20% |
| **Mild multifocal lymphoplasmacytic renal serositis** | | | 3/39 | | 2/19 | | | 0/10 | | | 0/6 | | | 1/1 | | | 0/2 | | 0/1 | 3/29 | 0/10 |
|  | | | 8% | | 11% | | | 0% | | | 0% | | | 100% | | | 0% | | 0% | 10% | 0% |
| **Mild multifocal renal cortical interstitial calcification** | | | 3/39 | | 2/19 | | | 0/10 | | | 0/6 | | | 1/1 | | | 0/2 | | 0/1 | 3/29 | 0/10 |
|  | | | 8% | | 11% | | | 0% | | | 0% | | | 100% | | | 0% | | 0% | 10% | 0% |
| **Mild multifocal lymphoplasmacytic peri-renal steatitis** | | | 5/39 | | 3/19 | | | 2/10 | | | 0/6 | | | 0/1 | | | 0/2 | | 0/1 | 5/29 | 0/10 |
|  | | | 13% | | 16% | | | 20% | | | 0% | | | 0% | | | 0% | | 0% | 17% | 0% |
| **Mild focal renal cortical fibrosis** | | | 1/39 | | 0/19 | | | 0/10 | | | 0/6 | | | 0/1 | | | 0/2 | | 1/1 | 1/29 | 0/10 |
|  | | | 3% | | 0% | | | 0% | | | 0% | | | 0% | | | 0% | | 100% | 3% | 0% |
| **Small numbers of mineral deposits in the renal tubular lumen** | | | 7/39 | | 3/19 | | | 3/10 | | | 1/6 | | | 0/1 | | | 0/2 | | 0/1 | 5/29 | 2/10 |
|  | | | 18% | | 16% | | | 30% | | | 17% | | | 0% | | | 0% | | 0% | 17% | 20% |
| **Mild multifocal follicular lymphoid hyperplasia at the renal cortico-medullary junction** | | | 11/39 | | 5/19 | | | 3/10 | | | 3/6 | | | 0/1 | | | 0/2 | | 0/1 | 9/29 | 2/10 |
|  | | | 28% | | 26% | | | 30% | | | 50% | | | 0% | | | 0% | | 0% | 31% | 20% |
| **Mild multifocal lymphoplasmacytic and eosinophilic cystitis** | | | 10/36 | | 2/19 | | | 5/10 | | | 1/3 | | | 0/1 | | | 2/2 | | 0/1 | 8/27 | 2/9 |
|  | | | 28% | | 11% | | | 50% | | | 33% | | | 0% | | | 100% | | 0% | 30% | 22% |
| **Mild multifocal submucosal bladder calcification** | | | 1/36 | | 0/19 | | | 1/10 | | | 0/3 | | | 0/1 | | | 0/2 | | 0/1 | 0/27 | 1/9 |
|  | | | 3% | | 0% | | | 10% | | | 0% | | | 0% | | | 0% | | 0% | 0% | 11% |
| **Mild to moderate focal umbilical arterial luminal calcification** | | | 3/36 | | 3/19 | | | 0/10 | | | 0/3 | | | 0/1 | | | 0/2 | | 0/1 | 2/27 | 1/9 |
|  | | | 8% | | 16% | | | 0% | | | 0% | | | 0% | | | 0% | | 0% | 7% | 11% |
| **Small amount of haematoidin in umbilical artery** | | | 4/36 | | 4/19 | | | 0/10 | | | 0/3 | | | 0/1 | | | 0/2 | | 0/1 | 2/27 | 2/9 |
|  | | | 11% | | 21% | | | 0% | | | 0% | | | 0% | | | 0% | | 0% | 7% | 22% |
| **Mild to moderate multifocal lymphoplasmacytic and eosinophilic skeletal myositis** | | | 7/37 | | 1/18 | | | 2/10 | | | 3/4 | | | 0/1 | | | 1/2 | | 0/2 | 7/28 | 0/9 |
|  | | | 19% | | 6% | | | 20% | | | 75% | | | 0% | | | 50% | | 0% | 25% | 0% |
| **Mild focal acute skeletal haemorrhage** | | | 1/37 | | 0/18 | | | 1/10 | | | 0/4 | | | 0/1 | | | 0/2 | | 0/2 | 0/28 | 1/9 |
|  | | | 3% | | 0% | | | 10% | | | 0% | | | 0% | | | 0% | | 0% | 0% | 11% |
| **Mild multifocal lymphoplasmacytic and eosinophilic diaphragmatic serositis** | | | 4/36 | | 1/19 | | | 2/9 | | | 1/3 | | | 0/1 | | | 0/2 | | 0/2 | 3/27 | 1/9 |
|  | | | 11% | | 5% | | | 22% | | | 33% | | | 0% | | | 0% | | 0% | 11% | 11% |
| **Mild multifocal lymphoplasmacytic and eosinophilic diaphragmatic myositis** | | | 3/36 | | 0/19 | | | 2/9 | | | 1/3 | | | 0/1 | | | 0/2 | | 0/2 | 3/27 | 0/9 |
|  | | | 8% | | 0% | | | 22% | | | 33% | | | 0% | | | 0% | | 0% | 11% | 0% |
| **Mild multifocal diaphragmatic interstitial fibrosis** | | | 1/36 | | 0/19 | | | 0/9 | | | 1/3 | | | 0/1 | | | 0/2 | | 0/2 | 1/27 | 0/9 |
|  | | | 3% | | 0% | | | 0% | | | 33% | | | 0% | | | 0% | | 0% | 4% | 0% |
| **Mild multifocal diaphragmatic muscle fiber atrophy** | | | 1/36 | | 0/19 | | | 0/9 | | | 1/3 | | | 0/1 | | | 0/2 | | 0/2 | 1/27 | 0/9 |
|  | | | 3% | | 0% | | | 0% | | | 33% | | | 0% | | | 0% | | 0% | 4% | 0% |
| **Severe locally extensive granulomatous fungal dermatitis and cellulitis (consistent with lobomycosis)** | | | 1/40 | | 0/19 | | | 0/10 | | | 0/6 | | | 0/1 | | | 0/2 | | 1/2 | 1/30 | 0/10 |
|  | | | 3% | | 0% | | | 0% | | | 0% | | | 0% | | | 0% | | 50% | 3% | 0% |
| **Mild multifocal lymphoplasmacytic dermatitis** | | | 2/40 | | 0/19 | | | 1/10 | | | 0/6 | | | 0/1 | | | 0/2 | | 1/2 | 2/30 | 0/10 |
|  | | | 5% | | 0% | | | 10% | | | 0% | | | 0% | | | 0% | | 50% | 7% | 0% |
| **Mild multifocal lymphoplasmacytic cellulitis** | | | 2/40 | | 2/19 | | | 0/10 | | | 0/6 | | | 0/1 | | | 0/2 | | 0/2 | 1/30 | 1/10 |
|  | | | 5% | | 11% | | | 0% | | | 0% | | | 0% | | | 0% | | 0% | 3% | 10% |
| **Moderate multifocal pseudoacanthomatous epidermal hyperplasia** | | | 3/40 | | 1/19 | | | 1/10 | | | 1/6 | | | 0/1 | | | 0/2 | | 0/2 | 3/30 | 0/10 |
|  | | | 8% | | 5% | | | 10% | | | 17% | | | 0% | | | 0% | | 0% | 10% | 0% |
| **Mild multifocal optic nerve melanosis** | | | 1/35 | | 0/16 | | | 0/9 | | | 1/6 | | | 0/1 | | | 0/1 | | 0/2 | 1/25 | 0/10 |
|  | | | 3% | | 0% | | | 0% | | | 17% | | | 0% | | | 0% | | 0% | 5% | 0% |
| **Mild to moderate multifocal optic peri-neural mineralization** | | | 4/35 | | 1/16 | | | 1/9 | | | 2/6 | | | 0/1 | | | 0/1 | | 0/2 | 3/25 | 1/10 |
|  | | | 11% | | 6% | | | 11% | | | 33% | | | 0% | | | 0% | | 0% | 12% | 10% |
| **Mild multifocal lymphoplasmacytic optic peri-neuritis** | | | 2/35 | | 0/16 | | | 0/9 | | | 2/6 | | | 0/1 | | | 0/1 | | 0/2 | 1/25 | 1/10 |
|  | | | 6% | | 0% | | | 0% | | | 33% | | | 0% | | | 0% | | 0% | 4% | 10% |
| **Mild multifocal lymphoplasmacytic optic scleritis** | | | 4/35 | | 3/16 | | | 0/9 | | | 1/6 | | | 0/1 | | | 0/1 | | 0/2 | 4/25 | 0/10 |
|  | | | 11% | | 19% | | | 0% | | | 17% | | | 0% | | | 0% | | 0% | 16% | 0% |
