# Supplementary material for: A Systematic Health Assessment of Indian Ocean Bottlenose (Tursiops aduncus) and Indo-Pacific Humpback (Sousa plumbea) Dolphins Incidentally Caught in Shark Nets off the KwaZulu-Natal Coast, South Africa
Source: PLoS One. 2014 Sep 9;9(9):e107038. doi: 10.1371/journal.pone.0107038 (PMC4159300; doi:10.1371/journal.pone.0107038)
Supplement: Table S3 — Common pathology observed in Tursiops aduncus and associations with sex, age and region. (DOCX) [file pone.0107038.s003.docx]

# Table S3: Common pathology observed in Tursiops aduncus and associations with sex, age and region

| **Lesion/abnormality** | **Total (%)** | **Sex (*n*)** | | | **Age class(*n*)** | | | | **Region (*n*)** | | |
| --- | --- | --- | --- | --- | --- | --- | --- | --- | --- | --- | --- |
|  |  | **Female** | **Male** | ***P**** | **<2 y** | **2-12 y** | **>12 y** | ***P*** | **North** | **South** | ***P*** |
| Pneumonia (all forms) | 91 | 23/24 | 9/11 | 0.227 | 17/19 | 10/10 | 5/6 | 0.565 | 22/25 | 10/10 | 0.542 |
| Bronchopneumonia | 20 | 5/24 | 2/11 | 1.000 | 5/19 | 1/10 | 1/6 | 0.844 | 4/25 | 3/10 | 0.381 |
| Interstitial pneumonia | 63 | **18/24** | **4/11** | **0.057** | 10/19 | 8/10 | 4/6 | 0.365 | 14/25 | 8/10 | 0.259 |
| Broncho-interstitial pneumonia | 26 | 5/24 | 4/11 | 0.416 | 6/19 | 2/10 | 1/6 | 0.770 | 8/15 | 1/10 | 0.235 |
| Pulmonary parasites | 17 | 4/24 | 2/11 | 1.000 | 5/19 | 1/10 | 0/6 | 0.423 | 4/25 | 2/10 | 1.000 |
| Pleuritis | 29 | **4/24** | **6/11** | **0.041** | 6/19 | 4/10 | 0/6 | 0.246 | 7/25 | 3/10 | 1.000 |
| Bronchiolar mucosal calcification | 83 | 21/24 | 8/11 | 0.352 | 16/19 | 8/10 | 5/6 | 1.000 | 21/25 | 8/10 | 1.000 |
| Pulmonary anthracosis | 6 | 1/24 | 1/11 | 0.536 | **0/19** | **0/10** | **2/6** | **0.025** | 1/25 | 1/10 | 0.496 |
| Enteritis | 71 | 18/24 | 7/11 | 0.689 | 9/19 | 10/10 | 6/6 | **0.002** | 19/25 | 6/10 | 0.421 |
| Gastritis (all compartments) | 71 | 17/24 | 7/10 | 1.000 | 10/19 | 8/9 | 6/6 | **0.034** | 16/24 | 8/10 | 0.683 |
| Gastritis compartments 1 & 2 | 68 | 16/24 | 7/10 | 1.000 | 9/19 | 8/9 | 6/6 | **0.017** | 16/24 | 7/10 | 1.000 |
| Gastritis compartment 3 | 67 | 10/14 | 4/7 | 0.638 | 6/11 | 4/6 | 4/4 | 0.391 | 9/14 | 5/7 | 1.000 |
| Parasitic nodules (all compartments) | 29 | 8/24 | 4/10 | 1.000 | 5/19 | 5/9 | 2/6 | **0.097** | 6/24 | 6/10 | 0.431 |
| Parasitic nodules compartments 1 & 2 | 6 | 3/24 | 0/10 | 1.000 | 2/19 | 1/9 | 0/6 | 1.000 | 2/24 | 1/10 | 0.508 |
| Parasitic nodules compartment 3 | 48 | 6/14 | 4/7 | 0.659 | 4/11 | 4/6 | 2/4 | 0.620 | 6/14 | 4/7 | 0.659 |
| Pyloric mucosal calcification | 24 | 4/14 | 1/7 | 0.624 | 2/11 | 2/6 | 1/4 | 0.805 | 3/14 | 2/7 | 1.000 |
| Periportal hepatitis | 60 | 16/24 | 5/11 | 0.283 | 11/19 | 5/10 | 5/6 | 0.465 | 17/25 | 4/10 | 0.151 |
| Hepatic serositis | 26 | 4/24 | 5/11 | 0.103 | 15/4 | 4/10 | 1/6 | 0.568 | 8/25 | 1/10 | 0.235 |
| Periportal fibrosis | 26 | 6/24 | 3/11 | 1.000 | 2/19 | 3/10 | 4/6 | **0.020** | 6/25 | 3/10 | 0.694 |
| Hepatic trematode eggs | 9 | 2/24 | 1/11 | 1.000 | 0/19 | 1/10 | 2/6 | **0.044** | 2/25 | 1/10 | 1.000 |
| Bile ductule hyperplasia | 43 | 9/24 | 6/11 | 0.467 | 7/19 | 3/10 | 5/6 | 0.109 | 10/25 | 5/10 | 0.712 |
| Splenic filamentous peritonitis | 49 | 11/24 | 6/11 | 0.725 | 7/19 | 6/10 | 4/6 | 0.345 | 9/25 | 8/10 | **0.027** |
| Splenic serositis | 31 | 4/24 | 7/11 | **0.015** | 4/19 | 4/10 | 3/6 | 0.328 | 7/25 | 4/10 | 0.689 |
| Cervical lymph node serositis | 29 | 4/23 | 6/11 | **0.045** | 4/18 | 3/10 | 3/6 | 0.405 | 6/24 | 4/10 | 0.431 |
| Mesenteric lymph node serositis | 44 | 9/24 | 6/10 | 0.276 | 5/19 | 5/10 | 5/5 | **0.009** | 11/24 | 4/10 | 1.000 |
| Lung marginal lymph node serositis | 41 | 8/18 | 3/9 | 0.692 | 6/13 | 4/8 | 1/6 | 0.525 | 6/19 | 5/8 | 0.206 |
| Marginal lymph node anthracosis | 11 | 2/18 | 1/9 | 1.000 | 0/13 | 2/8 | 1/6 | 0.124 | 1/19 | 2/8 | 0.201 |
| Endometritis | 42 | N/A | N/A | N/A | 4/13 | 2/7 | 4/4 | **0.044** | 7/16 | 3/8 | 1.000 |
| Metritis | 21 | N/A | N/A | N/A | 2/13 | 0/7 | 3/4 | **0.019** | 3/16 | 2/8 | 1.000 |
| Oophoritis | 17 | N/A | N/A | N/A | 1/13 | 2/7 | 1/4 | 0.344 | 3/16 | 1/8 | 1.000 |
| Mastitis | 43 | N/A | N/A | N/A | 2/3 | 1/2 | 0/2 | 0.657 | 3/6 | 0/1 | 1.000 |
| Mammary *corpora amylacea* | 43 | N/A | N/A | N/A | 0/3 | 2/2 | 1/2 | 0.143 | 2/6 | 1/1 | 0.429 |
| Testicular serositis | 30 | N/A | N/A | N/A | 1/5 | 1/3 | 1/2 | 1.000 | 3/8 | 0/2 | 1.000 |
| Endo-, myo- and epicarditis | 57 | 14/24 | 6/11 | 1.000 | 11/19 | 8/10 | 1/6 | **0.060** | 14/25 | 6/10 | 1.000 |
| Cardiac fibrosis | 26 | 5/24 | 4/11 | 0.416 | 0/19 | 4/10 | 5/6 | **0.001** | 7/25 | 2/10 | 1.000 |
| Meningoencephalitis | 44 | 4/11 | 3/5 | 0.596 | 3/9 | 4/6 | 0/1 | 0.302 | 5/8 | 2/8 | 0.315 |
| Myositis | 19 | 5/21 | 1/11 | 0.637 | 1/18 | 2/10 | 3/4 | **0.007** | 6/23 | 0/9 | 0.150 |
| Combined serositis | 74 | 16/24 | 10/11 | 0.217 | 11/19 | 9/10 | 6/6 | **0.092** | 20/25 | 6/10 | 0.393 |
| Abdominal serositis | 57 | 12/24 | 8/11 | 0.281 | 7/19 | 7/10 | 6/6 | **0.013** | 15/25 | 5/10 | 0.712 |
| Thoracic serositis | 51 | 10/24 | 8/11 | 0.146 | 10/19 | 6/10 | 2/6 | 0.665 | 13/25 | 5/10 | 1.000 |

* statistically significant results (p<0.100) in bold.
